# Tumorigenic effects of human mesenchymal stromal cells and fibroblasts on bladder cancer cells

**DOI:** 10.3389/fonc.2023.1228185

**Published:** 2023-09-13

**Authors:** Lucie M. Frerichs, Bastian Frerichs, Patrick Petzsch, Karl Köhrer, Joachim Windolf, Bernd Bittersohl, Michèle J. Hoffmann, Vera Grotheer

**Affiliations:** ^1^ Department of Orthopedics and Trauma Surgery, Medical Faculty and University Hospital Düsseldorf, Heinrich-Heine-University, Düsseldorf, Germany; ^2^ Biological and Medical Research Center (BMFZ), Heinrich-Heine-University, Düsseldorf, Germany; ^3^ Department of Urology, Medical Faculty and University Hospital Düsseldorf, Heinrich-Heine-University, Düsseldorf, Germany

**Keywords:** tumor microenvironment, bladder cancer, mesenchymal stem cells, fibroblasts, cisplatin resistance, CD44, CXCR-4

## Abstract

**Background:**

Patients with muscle-invasive bladder cancer face a poor prognosis due to rapid disease progression and chemoresistance. Thus, there is an urgent need for a new therapeutic treatment. The tumor microenvironment (TME) has crucial roles in tumor development, growth, progression, and therapy resistance. TME cells may also survive standard treatment of care and fire up disease recurrence. However, whether specific TME components have tumor-promoting or tumor-inhibitory properties depends on cell type and cancer entity. Thus, a deeper understanding of the interaction mechanisms between the TME and cancer cells is needed to develop new cancer treatment approaches that overcome therapy resistance. Little is known about the function and interaction between mesenchymal stromal cells (MSC) or fibroblasts (FB) as TME components and bladder cancer cells.

**Methods:**

We investigated the functional impact of conditioned media (CM) from primary cultures of different donors of MSC or FB on urothelial carcinoma cell lines (UCC) representing advanced disease stages, namely, BFTC-905, VMCUB-1, and UMUC-3. Underlying mechanisms were identified by RNA sequencing and protein analyses of cancer cells and of conditioned media by oncoarrays.

**Results:**

Both FB- and MSC-CM had tumor-promoting effects on UCC. In some experiments, the impact of MSC-CM was more pronounced. CM augmented the aggressive phenotype of UCC, particularly of those with epithelial phenotype. Proliferation and migratory and invasive capacity were significantly increased; cisplatin sensitivity was reduced. RNA sequencing identified underlying mechanisms and molecules contributing to the observed phenotype changes. NRF2 and NF-κB signaling was affected, contributing to improved cisplatin detoxification. Likewise, interferon type I signaling was downregulated and regulators of epithelial mesenchymal transition (EMT) were increased. Altered protein abundance of CXCR4, hyaluronan receptor CD44, or TGFβ-signaling was induced by CM in cancer cells and may contribute to phenotypical changes. CM contained high levels of CCL2/MCP-1, MMPs, and interleukins which are well known for their impact on other cancer entities.

**Conclusions:**

The CM of two different TME components had overlapping tumor-promoting effects and increased chemoresistance. We identified underlying mechanisms and molecules contributing to the aggressiveness of bladder cancer cells. These need to be further investigated for targeting the TME to improve cancer therapy.

## 1 Introduction

Urothelial carcinoma of the bladder cancer (UC) is the 10th most commonly diagnosed cancer worldwide, with 573,278 new cases in 2020 and a higher prevalence in men (1). In total, 25% of UC patients suffer from muscle-invasive bladder cancer (MIBC) and face a poor prognosis often due to rapid local and systemic progression. The standard of care is radical cystectomy accompanied by (neo-) adjuvant chemotherapy or the recently approved modern immunotherapy with immune checkpoint inhibitors. However, the 5-year survival rate is below 50% due to therapy resistance and disease progress (2–4).

Therefore, new therapeutic options are urgently needed to improve bladder cancer treatment. Involvement of the tumor environment (TME) in treatment resistance and how this could be targeted is one current research focus. Fibroblasts (FB), mesenchymal stromal cells (MSC), pericytes, endothelial cells, and immune cells, as functional components of the TME, can affect cancer initiation, angiogenesis, invasion, and metastasis (5–8). TME cells may also be involved in the development of chemoresistance, e.g., toward cisplatin in esophageal cancer *via* PAI-1 secretion (9) and in head and neck cancers (10). For bladder cancer, increasing evidence also suggests that, e.g., cancer-associated fibroblasts (CAF) may affect treatment response by direct cellular interaction or cytokine signaling. Treatment of surviving stromal cells may also promote the occurrence of clinical relapse (11, 12). Thus, development of new therapeutic approaches targeting not only cancer but also TME cells came into focus, which demands for a detailed understanding of the mechanisms and molecules underlying the interaction between cancer and TME cells. The characterization of TME cells and their effects on cancer cells has been challenging due to their complex interactions, heterogeneity of, e.g., FB and tissue dependency. Pan cancer secretome *in*-*silico* analyses further supported the tissue and cell type dependency of mechanism and mediating molecules, indicating that these need to be investigated in detail in the respective tissue-dependent context and that results from other cancer entities cannot simply be transferred (13). Concurringly, both tumor-inhibiting or tumor-promoting properties of TME cells have been described (14–19). Thus, we aimed in this study to decipher mechanisms and molecules underlying the interaction of TME and bladder cancer cells to identify players and new therapeutic targets.

In bladder cancer, recently defined molecular subtypes that are associated with differences in patients’ prognosis and chemotherapy response differ in the extent of stromal differentiation and abundance of stromal cell types (20). Different FB subpopulations have been characterized in bladder cancer tissues that were also associated with patient’s survival and histopathology, clearly indicating the clinical significance of TME cells in bladder cancer and the need for a further detailed investigation (21).

Since FB are an essential component of the connective tissue (22, 23), which surrounds the bladder in the form of the lamina propria (24), this cell type is one source of stromal cells in the bladder TME. FB are highly proliferative and have multilineage differentiation potential and immunomodulatory features (25, 26). FB regulate inflammation, wound healing processes (27), induce angiogenesis (28, 29) and are an essential source of extracellular matrix (ECM)-degrading proteins such as matrix metalloproteinases (MMP) (30). It is known that FB can promote the growth and progression of cancer and are therefore considered as new targets for cancer therapies (23).

Since CAFs may also originate from tumor-induced differentiation of MSC (31), we considered MSC as a key population of TME cells. Concurringly, MSC are currently intensively investigated in the cancer context. In benign tissues, stromal MSC are responsible for tissue homeostasis and for the continuous replacement of pathophysiologically altered or destroyed cells. MSC are found in almost every tissue and have multipotent differentiation potential and immunomodulatory features as well as modulate neovascularization and paracrine effects (32–35). However, MSC differ quantitatively and qualitatively depending on the tissue origin and body site—for example, adipose-derived stromal cells contain comparatively more colony-forming units (stem cells) and have improved immunomodulating properties than bone marrow-derived stromal cells (36–38). With regard to bladder cancer, mesenchymal adipose-derived stromal cells are of particular interest since the bladder is surrounded by adipose tissue and MSC had been also isolated from the human bladder earlier (24, 39, 40). Generally, MSC have been shown to mediate pro-tumorigenic effects and chemoresistance in breast cancer (41), colon, and skin (42). By induction of metabolic changes in cancer cells and production of energy metabolites, they may provide metabolic support to the fast cycling of cancer cells. They further contribute to chemoresistance and immunosuppression by their immune-modulating properties (43). The latter may be relevant not only for immune escape of cancer cells but also for treatment response to recently approved immunotherapy with immune checkpoint inhibitors for bladder cancers (40). Thus, a detailed investigation of MSC effects on bladder cancer cells is urgently needed since they may significantly limit the treatment response to all current standard-of-care treatments of UC. However, a systematic review published in 2021 summarized both the inhibitory and augmenting effects of MSC on different cancer cell entities, indicating that the effects of MSC are cell type-specific and need to be investigated in individual cancer types (44). With regard to genitourinary cancers, prostate cancer cells were intensively analyzed. For bladder cancer, this review comprised only data from one bladder cancer cell line (T24). To date, the current knowledge of the impact of the TME, particularly of MSC and FB on bladder cancer cells is still limited (45). Particularly, the functional impact of MSC on bladder cancer cells has only been investigated in a few new *in vitro* studies on UC cell lines 5637 and HT-1376 with partially contradictory results. Therefore, the present study aimed to investigate the cellular and molecular effects of multi-donor pooled conditioned media (CM) from MSC or FB on tumorgenicity, epithelial mesenchymal transition (EMT), and migration and invasion capacity of urothelial carcinoma cell lines (UCC). Furthermore, analysis of secreted proteins and RNA sequencing analysis were performed with UCC treated with MSC- and FB-CM to analyze the underlying mechanisms and players that could be targeted in the future.

## 2 Materials and methods

### 2.1 Standard cell cultivation

MSC and FB were cultivated in Dulbecco’s Modified Eagle’s Medium (DMEM; 4.5 g/L glucose) with 2 mM α-glutamine, 100 U/mL penicillin, 100 µg/mL streptomycin, and 10% fetal bovine serum (FBS) (Biochrom, Berlin, Germany). The cells were maintained at 37°C in a humidified atmosphere containing 5% CO_2_.

### 2.2 Isolation and culture of MSC

MSC were isolated following an established protocol from human abdominoplasty (46). Briefly, adipose tissue was cut into small pieces (5 mm^2^) and digested with collagenase solution type I (type: CLS 255 U/mg; 0.2%) at 37°C for 45 min with constant shaking. The ratio of tissue to enzyme was 1:2. After filtration (100 µm), the suspension was centrifuged at 300*g* for 10 min. The fat layer was removed, and the cell suspension was centrifuged again at 300*g* for 10 min. After resuspension, the cells were seeded in cell culture flasks and cultured in a standard cell culture medium (as mentioned above). Validation of the MSC culture technique by the analysis of characteristic marker expression was reported earlier (47).

### 2.3 Isolation of FB

FB were isolated as described previously (48). Briefly, skin samples were cut into small pieces (5 mm^2^) and digested overnight with 0.2% dispase II solution. The samples were treated with 0.2% collagenase type I (type: CLS 255 U/mg) buffer (1 mM CaCl, 5 mM glucose, 0.1 M HEPES, 0.12 M NaCl, and 50 mM KCl in aqua dest.) for 2 h at 37°C in a shaking water bath to release the cells from the tissue matrix. Following digestion, the suspension was passed through a filter (100 µm), washed with PBS, and centrifuged at 300*g* for 5 min. The cell pellet was resuspended in a standard culture medium and incubated at 37°C, 5% CO_2_.

### 2.4 Production of conditioned media

MSC and FB were seeded in T175 cm^2^ cell culture flasks in 35 mL standard cell culture medium. MSC were plated in a seeding density of 9 × 10^5^ and FB in a cell concentration of 7 × 10^5^. After 72 h, media from six different donors in cell culture passages 3–8 were collected and sterile-filtrated (0.2 µm). The CM was aliquoted and frozen at -80°C until pooling on a parity basis and usage. For further experiments, CM of six different donors were pooled prior to application to UC cell lines.

### 2.5 Urothelial carcinoma cell lines and culture

Three urothelial cancer cell lines (UCC), namely, UMUC-3 (RRID : CVCL_1783, male), BFTC905 (RRID : CVCL_1083, female), and VMCUB-1 (RRID : CVCL_1786, male), were selected since they originate from rather progressive tumors and represent the heterogeneity of the disease. The characteristics and genomics of commercially available UC lines were summarized in (49, 50). Since we aimed to determine the TME effects on epithelial–mesenchymal transition (EMT), we selected BFCT905 and VMCUB-1 also due to their epithelial morphology, while UMUC-3 cells present a mesenchymal morphology. UCC were obtained from the DSMZ (Braunschweig, Germany) and Dr. H.B. Grossmann (Houston, TX, USA). These were cultured in DMEM (4.5 g/L glucose) with 2 mM α-glutamine and GlutaMAX™-Supplement, 10% FBS, 100 U/mL penicillin, and 100 µg/mL streptomycin at 37°C in a humidified atmosphere at 5% CO_2_. For the experiments, UCC were seeded in undiluted CM for 72 h. The exceptions were Western blot, gene expression analysis, and the additional invasion assay for BFTC-905. For these experiments, UCC were preincubated for 6 days in CM since we observed in the course of our study that certain effects took a longer incubation time to establish pronounced effects in UCC.

### 2.6 Cell viability assay

UCC were seeded with a standard cell culture medium or with CM. After 72 h, cell viability was determined using CellTiter-Blue (Promega, Madison, USA). CellTiter-Blue’s working solution was diluted in medium (1:20). CellTiter-Blue uses an indicator dye to measure the metabolic activity of cells, as indirect evidence for cell viability. After 1 h of incubation, fluorescence (540_Ex_/590_Em_) was measured in a 1420 Multilabel Counter (Victor^3^, Perkin Elmer). For the analysis of cisplatin resistance, UCC were seeded in standard cell culture medium or with CM for 24 h. Then, the respective cisplatin concentrations (0.4–6 µM) were added. After 72 h, cell viability assay was performed.

### 2.7 Migration assay

For the migration assay, ibidi™ inserts (Thistle Scientific Ltd., UK) were used. The ibidi inserts were placed in 12-well culture plates using sterile tweezers. Cell suspensions were prepared at 2.3 × 10^4^ cells/chamber for VMCUB-1, 2.4 × 10^4^ cells/chamber for UMUC-3, and 4 × 10^4^ cells/chamber for BFTC905. After 20 h of incubation with a standard cell culture medium or CM, the inserts were carefully removed and 1.5 mL conditioned media was applied. Photo-documentation was performed after 10 h and for BFTC905 after 12 h. Cell migration was assessed using ImageJ Freehand Selection Tools by measuring the area covered with the UCC. The results were compared with the 0-h time point. Every scratch was analyzed in duplicate. For each cell line, seven to nine differently conditioned media were used, each representing a pool of six donors.

### 2.8 Invasion assay

In total, 24 Transwell chambers (8 μm in pore size) were pre-coated with 20 µL Matrigel (Clontech, Mountain View, CA, USA) and incubated for 20 min (37°C, 5% CO_2_), followed by a further coating step with 20 µL Matrigel for 1 h. For VMCUB-1 and UMUC-3, 3 × 10^4^ cells in 100 μL Opti-MEM® Reduced-Serum medium were added to the upper chamber. For BFTC905, 5 × 10^4^ cells were seeded, and 500 μL of conditioned media was added into the lower chamber. Then, 20 h (VMCUB-1 and UMUC-3) to 24 h (BFTC905) later, non-adherent cells were cautiously removed using a cotton swab. BFTC905 were additionally preincubated with CM media (PCM) for 6 days before the experimental setup. The cells in the chambers were washed with PBS (4°C) and fixed for 10 min with ice-cold methanol and then stained with crystal violet for 25 min. After a further washing step and 20 min of drying time, the membrane was removed with a scalpel and embedded in xylol. The membrane was positioned on a microscope slide and coated with DePeX (Serva, Heidelberg, Germany). Three representative images of each membrane were taken with a Zeiss Axiovert 200. The area covered with invaded cells was analyzed using ImageJ, and an average value was calculated. For each cell line, nine differently conditioned media were analyzed, with each of them representing a pool of six donors.

### 2.9 Serpin E1/PAI-ELISA

UCC were treated for 6 days with a standard cell culture medium or with CM. After that, cell culture supernatants were diluted (1:3), and 100 µL of the respective samples was added in duplicate into 96 wells. Analysis was performed according to the manufacturer’s specifications (DuoSet ELISA, R&DSystems, DY1786MN, Minneapolis, MN 55413, USA). The optical density was determined using Victor X3 Multilabel-Reader (PerkinElmer, CT, USA).

### 2.10 Western blot analysis

To evaluate protein abundance, Western blot analysis was performed. After the UCC were treated for 6 days with a standard cell culture medium or CM, the protein concentration was analyzed with the Pierce BCA Protein Assay Kit (ThermoFisher Scientific, Darmstadt, Germany). For electrophoresis, 10 μg (SMAD4 and *α*-SMA) or 20 μg protein was mixed with 5 µL of Laemmli buffer (4 × Trisglycin-SDS sample buffer, 252 mmol Tris-HCl, pH 6.8; 40% glycerin; 8% SDS; 0.01% bromphenol blue + 20% mercaptoethanol), centrifuged (3,000*g*, 5 min at 4°C), denatured for 5 min at 95°C, and separated on 12% sodium dodecyl sulphate-polyacryl-amide gel (SDS-PAGE). The separated proteins were transferred using BioRad Trans-Blot Turbo to a nitrocellulose membrane (Roche, Mannheim, Germany). Antigens were detected with the following antibodies: E-cadherin (Abcam ab15148; 1:1,000, RRID : AB_301693), vimentin (Thermo Fisher Scientific MA5-14564; 1:1,000, RRID : AB_10981427), SMAD4 (Santa Cruz Technologies sc7966; 1:1,000, RRID : AB_627905), CXCR-4 (Thermo Fisher Scientific; PA5-19856; 1:1,000, RRID : AB_11152329), and *α*-SMA (Abcam ab7817; 1:3,000, RRID : AB_262054). Anti-rabbit or anti-mouse conjugated with horseradish peroxidase (HRP) served (1:1,000) as secondary antibodies, and according to the manufacturer’s guidance, the protein concentration was normalized to that of total protein. Western blots were visualized with ChemiDoc MP Imaging system and analyzed with Image Lab, version 6.0.1, build 34, 2017, standard edition, BioRad Laboratories.

### 2.11 Flow cytometer analysis of CD44 receptor protein

CD44 (BD Biosciences, catalog no.: 559942, RRID : AB_398683, Heidelberg, Germany) protein abundance in UCC was analyzed after UCC had been treated for 6 days with a standard cell culture medium or with CM with a flow cytometer (BD FACS Lyric, IC-Nr.: 87135) and BD FACSsuite Software, BD Biosciences, Heidelberg, Germany). An appropriate isotype-matched control antibody was used as control in all analyses. For the flow cytometer analysis, UCC were washed with PBS and then detached with a cell scraper. Afterward, these were washed again with CellWash^®^ (BD Biosciences, Heidelberg, Germany) containing 3% FBS and centrifuged at 300*g* for 5 min, the supernatant was removed, and then UCC were blocked in 50 µL FBS on ice for 20 min. The cells were then resuspended and stained for 30 min with fluorophore-conjugated antibodies (10%). After two further washing steps with CellWash + FBS (3%), the samples were analyzed.

### 2.12 E-cadherin and vimentin immunofluorescence

BTC905, VMCUB-1, and UMUC3 cells were pre-incubated with CM for 6 days and seeded on coverslips for 72 h with or without (w/wo) CM. The cells were washed twice with PBS and fixed with Roti-Histofix (4%). After a further washing step, anti-E-cadherin (final dilution 1:50, Abcam) and anti-vimentin (final dilution 1:200, Invitrogen) antibodies were applied in Triton X-100 with normal goat serum overnight at 4°C. The cells were rewashed and stained with secondary antibodies conjugated with Alexa-594 or Alexa-488 for 30 min at room temperature (RT). Finally, nuclear staining was performed with 0.1% 4′,6-diamidino-2-phenylindole (DAPI) for 15 min at RT. The cells were washed again, covered with Fluoromount, and examined microscopically with Zeiss Axiovert 200.

### 2.13 mRNA expression analysis

RNA was extracted from UCC treated w/wo CM for 6 days using the RNeasy Mini Kit according to the manufacturer’s specifications (Qiagen, Hilden, Germany). One microgram of RNA was reverse-transcribed into cDNA using the QuantiTect Reverse Transcription Kit (Qiagen), with an extended incubation time of 30 min at 42°C. Quantitative reverse transcription polymerase chain reaction (qRT-PCR) was performed with PowerUp™ SYBR® Green Master Mix (Applied Biosystems®, Dreieich, Germany) according to the manufacturer’s instructions on the StepOne Real-Time PCR System (Applied Biosystems®, Dreieich, Germany). RNA expression was measured using the primers given in **Additional File 1**. As housekeeping genes, TATA-box binding protein (*TBP*) and glyceraldehyde-3-phosphate-dehydrogenase (*GAPDH*) were used. qRT-PCR was performed using initial denaturation at 95°C for 2 min and 45 cycles of amplification, including denaturation at 95°C for 10 s, annealing and elongation for 30 s at 60°C, and a melting curve analysis.

### 2.14 Next-generation RNA sequencing

Total RNA was extracted from BFTC-905 and VMCUB-1 cells at 72 h after treatment with FB-CM or MSC-CM or DMEM as a control. RNA was extracted as described above. Qubit RNA HS Assays (Thermo Fisher Scientific, MA, USA) were used for RNA quantification. The RNA qualities were confirmed by capillary electrophoresis using the fragment analyzer with total RNA Standard Sensitivity Assays (Agilent Technologies, Santa Clara, CA, USA). Library preparation and next-generation sequencing were performed as described (51). Multigroup comparisons were calculated using the Empirical Analysis of DGE (version 1.1, cutoff = 5) after grouping of samples (three biological replicates each) according to their respective experimental conditions. FDR and Bonferroni correction were applied to adjust the *p*-values for multiple testing. A *p*-value of ≤0.05 was considered as significant. The cutoff for differential gene expression was set to 1.5-fold. Further analysis and data visualization were performed using Microsoft Excel and Graph Pad Prism 8. Venn diagrams were prepared with the online tool Venny 2.0 (52). GO group analysis was performed using the online tool DAVID (53).

### 2.15 Proteome profiler human XL arrays

MSC-conditioned media from two different donors and FB-conditioned media from two different donors were applied to the Proteome Profiler Human XL Oncology Array membranes according to the manufacturer’s instructions (R&D Systems, Minneapolis, MN, USA). Dot blots were visualized with the ChemiDoc MP Imaging system and analyzed with Image Lab, version 6.0.1, build 34, 2017, standard edition, from BioRad Laboratories. The value of the negative control was subtracted from all other values. Values were further normalized to the values of the reference spots.

### 2.16 Statistical analysis

The statistical analysis was carried out with GraphPad Prism 8.0 (GraphPad Software Inc., San Diego, CA, USA). Two-tailed Student’s *t*-test or two-way ANOVA was used, and a *p*-value less than 0.05 was considered significant. The values shown are mean ± standard deviation (SD).

## 3 Results

### 3.1 MSC- and FB-CM augmented cell proliferation and chemoresistance of UC cell lines

**Figure 1:**
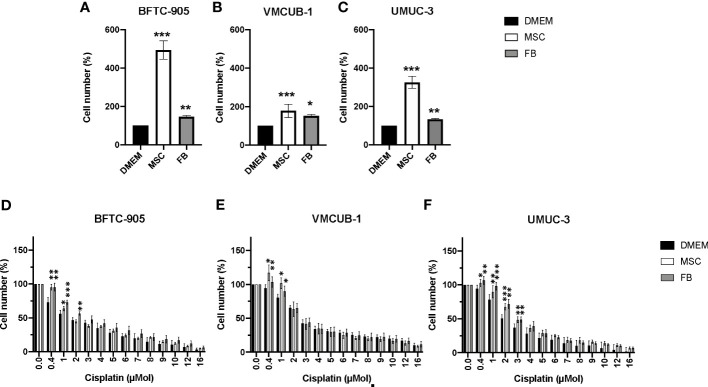
Effects of CM on cell proliferation and cisplatin sensitivity of UCC. **(A–C)** Cell viability was determined by CellTiter-Blue assay after treatment with CM for 72 h. DMEM -treated cells served as controls. The results of CM-treated cells were normalized to DMEM control cells. **(D–F)** Cell viability was again quantified by CellTiter-Blue assay after treatment with CM and the indicated doses of cisplatin for 72 h. Values were normalized to not cisplatin-treated cells set to 100%. Bars represent mean ± SD of the individual experiments indicated (*n* ≥ 6); *p ≤ 0.05, **p ≤ 0.01, ***p ≤ 0.001.

Initially, we determined whether CM from stromal cells (MSC or FB) had inhibitory or augmenting effects on the cellular growth of three different UC cell lines used as models for MIBC. Since we later aimed also to determine the effects on motility and EMT, we selected two cell lines with an epithelial phenotype, namely, BFTC-905 and VM-CUB1, compared to UMUC-3 cells with a mesenchymal phenotype. MSC-CM strongly augmented the cell growth of all three cell lines (**Figures 1A–C**). The FB-CM treatment was less effective, but still significant.

Since TME cells were earlier reported to promote chemoresistance, CM-treated UCC were additionally treated with cisplatin to analyze whether CM could affect the cisplatin sensitivity of treatment-naïve UCC. Generally, UCC treated with MSC- or FB-CM tolerated the sublethal cisplatin doses of 0.4–3 µM significantly better. Compared to the other two UCC (**Figures 1D, E**), mesenchymal UMUC-3 (**Figure 1F**) cells became slightly more cisplatin-resistant by conditioned media.

### 3.2 MSC- and FB-CM-enhanced invasive potential of UCC

**Figure 2:**
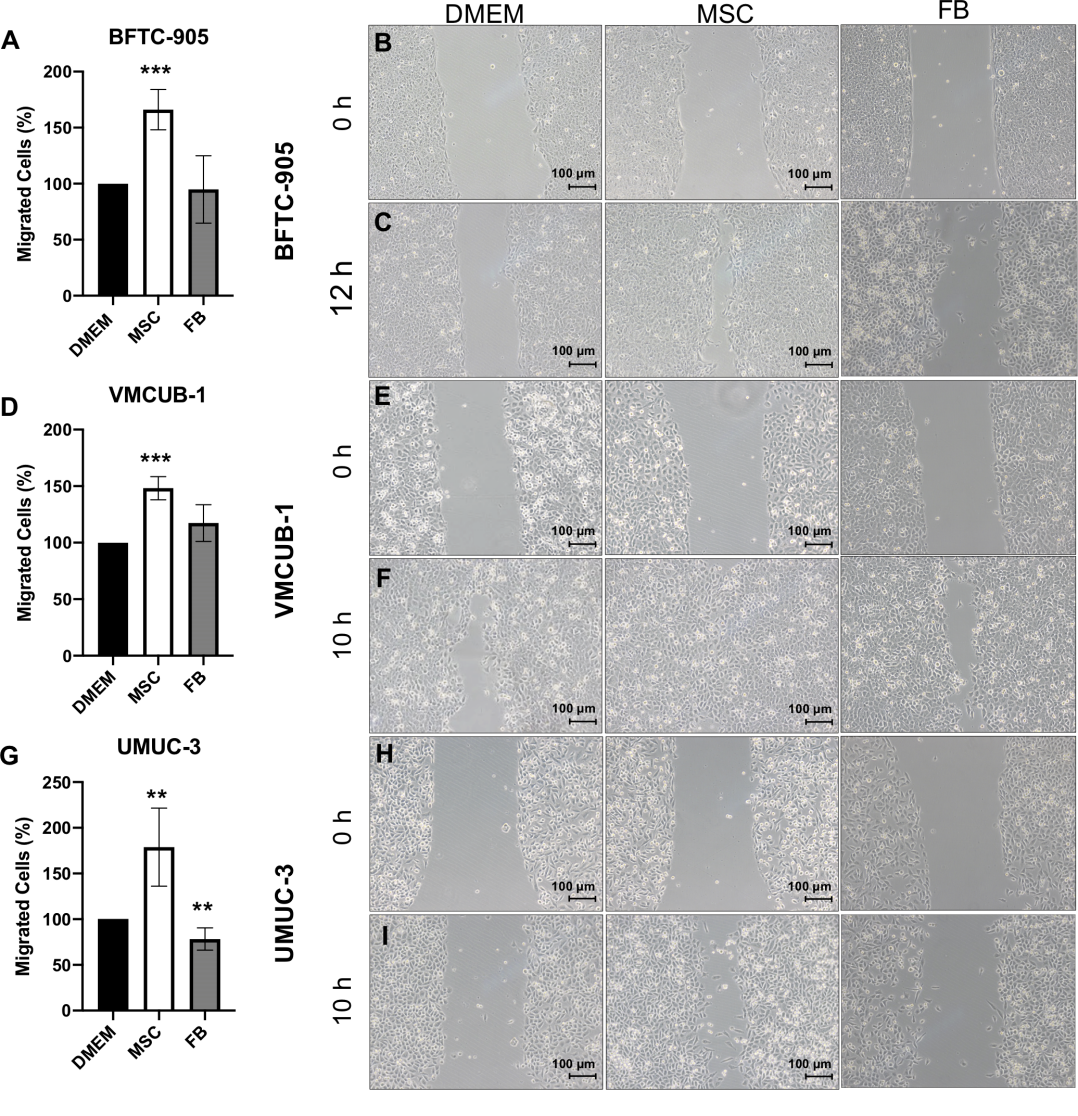
Effects of CM on the migration capacity of UCC. The migration capacity of UCC was measured at several time points by wound healing assay after UCC had been treated with CM for 72 h. Representative results at time point 10 h and for BFTC-905 at 12 h are displayed as photographs and bar column for BFTC-905 cells **(A–C)**, VMCUB-1 cells **(D–F)**, and UMUC-3 cells **(G–I)** (*n* ≥ 7). Bars represent mean ± SD of the individual experiments indicated (*n* ≥ 7); **p ≤ 0.01, ***p ≤ 0.001.

In the next step, we characterized the migratory and invasive potential of UCC treated with CM. Wound healing assays were performed to measure cell migration after 10 h (**Figures 2A–I**). The migration capacity was significantly increased by MSC-CM in all UCC, but not by FB-CM.

**Figure 3:**
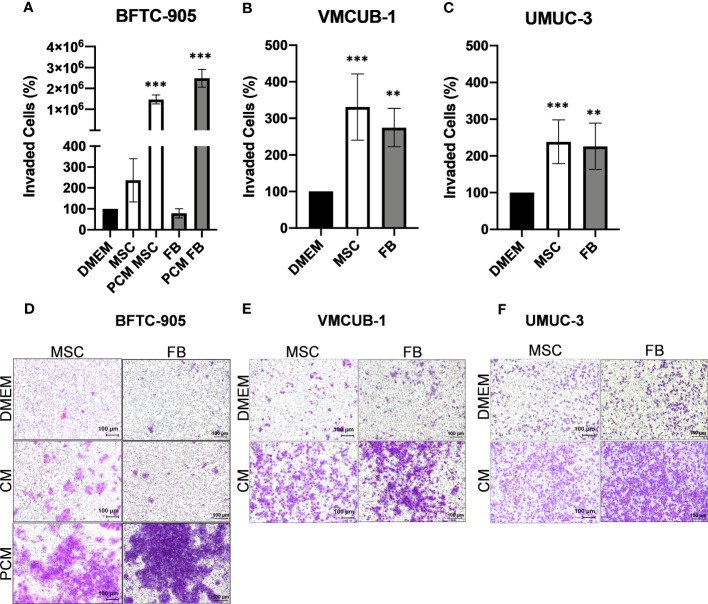
Effects of CM on the invasion capacity of UCC. The invasion capacity of UCC was analyzed by Boyden chamber assay after 20 h and for BFTC-905 after 24 h. Furthermore, BFTC-905 was pre-incubated with CM for 6 days. Bar graphs displaying the number of invaded cells **(A–C)** originating from images of stained invaded cells and counting **(D–F)**. Bars represent mean ± SD of the individual experiments indicated (*n* = 10). PCM: cells were pretreated with indicated CM prior to the invasion assay. **p ≤ 0.01, ***p ≤ 0.001.

Invasion potential (**Figure 3A–F**) was significantly increased by both CM types in VMCUB-1 and UMUC3 cells (**Figures 3B, C, E, F**). BFTC-905 cells became only more invasive when they were longer treated with CM (**Figures 3A, D**). Pretreatment for 3 days with CM (PCM) prior to the invasion assay also resulted in a highly significant increase in invasion of BFTC-905 cells.

**Figure 4:**
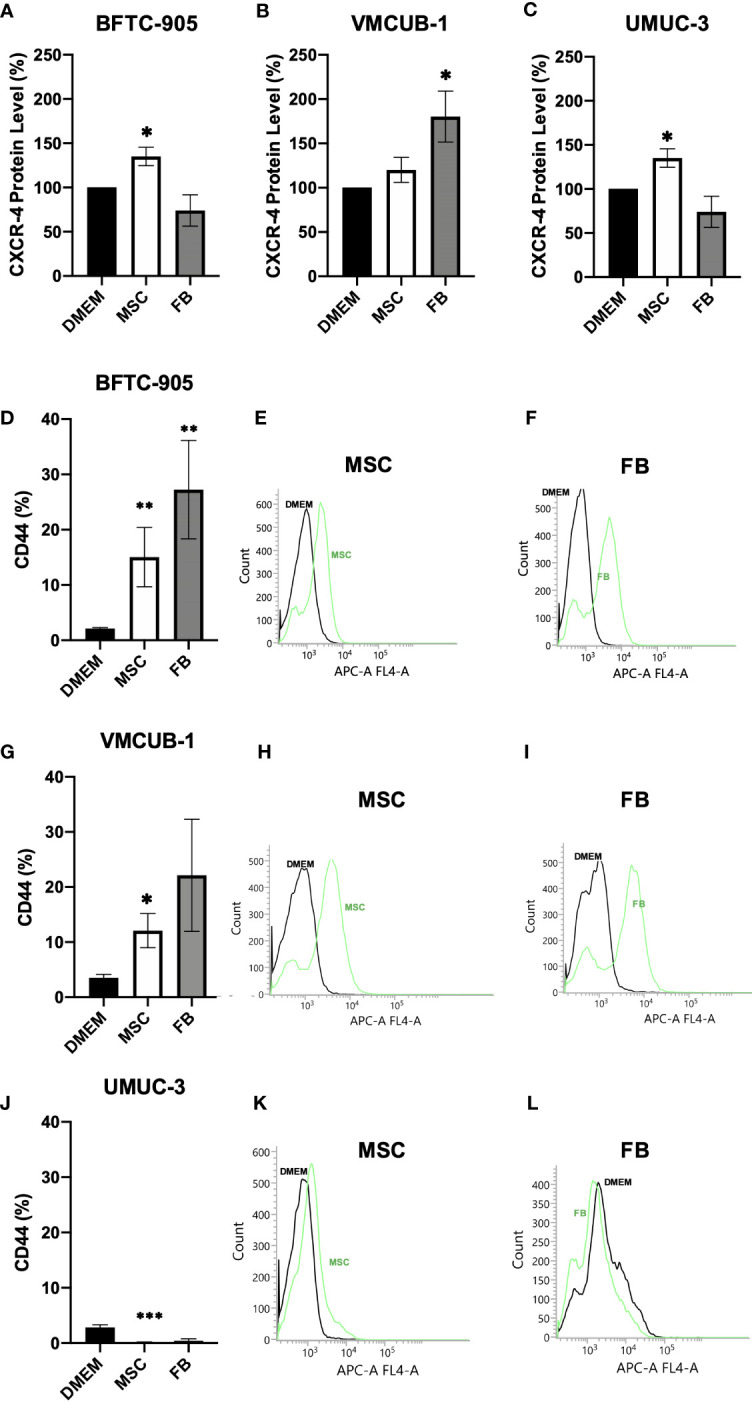
Protein analysis of CXRC4 and CD44. The protein levels of CXCR4 **(A–C)** and CD44 **(D–L)** of CM-treated (cells were pre-incubated for 6 days) and untreated UCC were determined by Western blot **(A–C)** and flow cytometer analysis **(D–L)** as shown by raw data histograms **(E, F, H, I, K, L)**. The protein levels were normalized to DMEM-treated controls. Bars represent mean ± SD of the individual experiments indicated (*n* ≥ 3); *p ≤ 0.05, **p ≤ 0.01, ***p ≤ 0.001.

Furthermore, the invasive potential of CM-treated UCC was characterized on the molecular level. The CXCR4 chemokine receptor protein level associated with migration (54) and metastatic homing (55) was enhanced by CM in a cell type-dependent manner. The levels were significantly increased in BFTC-905 and UMUC-3 by MSC-CM and in VMCUB-1 cells only by FB-CM (**Figures 4A–C**). Raw data from Western blot are shown in **Additional File 2**. As a receptor for hyaluronic acid, the CD44 levels were earlier shown to correlate with poor prognosis and metastatic potential (56) and were strongly elevated by MSC- and FB-CM treatment in both UCC with epithelial morphology, BFTC-905, and VMCUB-1 (**Figures 4D–L**). Mesenchymal UMUC-3 cells responded rather with decreased CD44 levels to CM.

**Figure 5:**
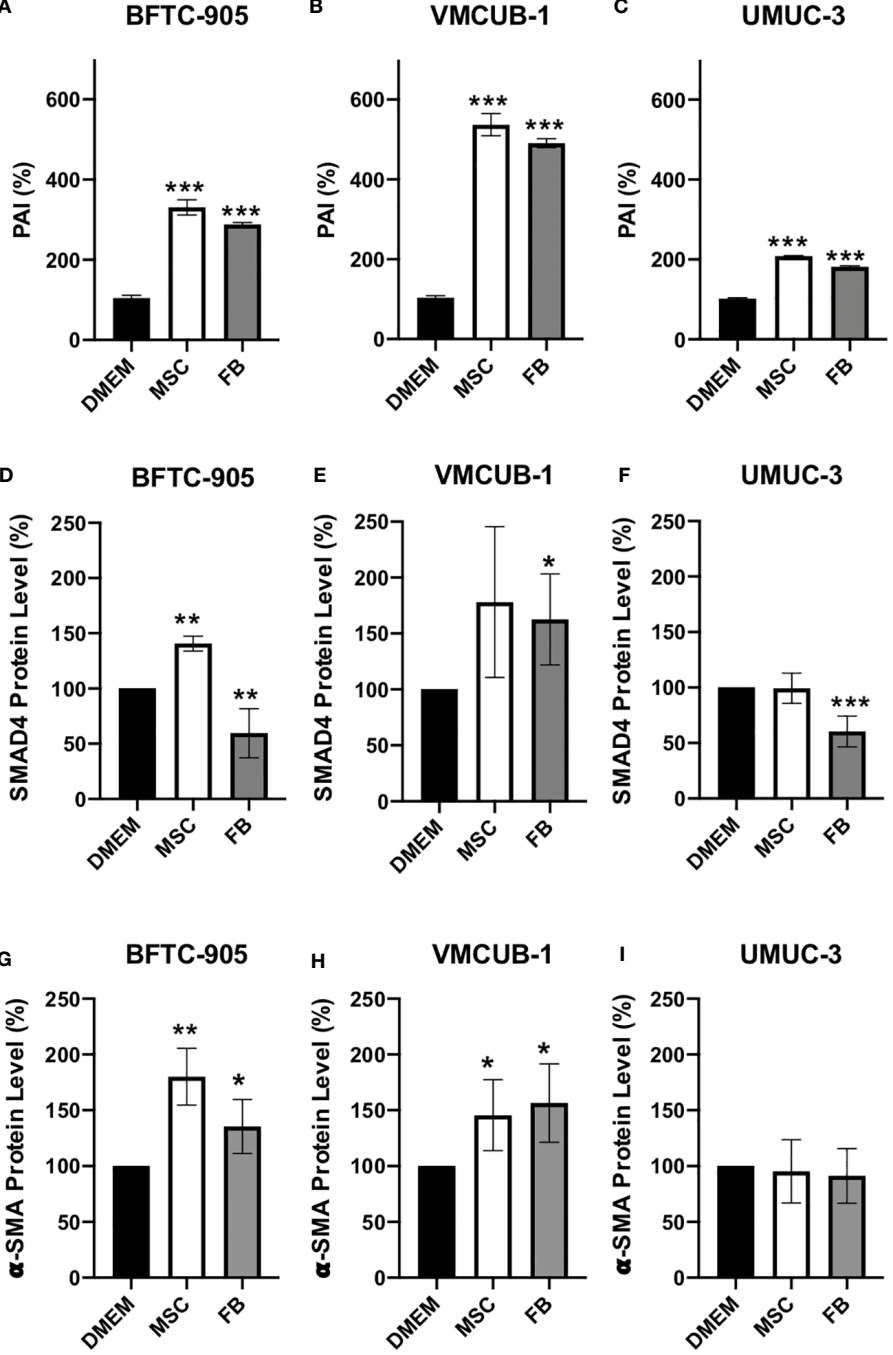
Protein analysis of PAI1, SMAD4, and α-SMA. Secreted PAI1 protein was quantified by ELISA assay **(A–C)**. The protein levels of SMAD4 **(D–F)** and α-SMA **(G–I)** of CM-treated and untreated cells (prior analysis UCC were pre-incubated for 6 days with CM) were determined by Western blot analysis. The protein levels were normalized to DMEM-treated controls. Bars represent mean ± SD of the individual experiments indicated (*n* = 3); *p ≤ 0.05, **p ≤ 0.01, ***p ≤ 0.001.

Since EMT is a known process underlying tumor invasion and metastasis that can be regulated by TGF-β signaling, we analyzed its downstream factors. To this end, we performed ELISA assays for SERPINE1/PAI1 (**Figures 5A–C**). Western blot analysis was performed for SMAD4- and α-SMA-protein levels (**Figures 5D–I**). PAI1 is predominantly expressed when TGF-β is activated and can be used as a marker for TGF-β activity (57). Secreted PAI1 levels were significantly increased in all conditions. Another downstream factor of TGF-β signaling is SMAD4, which mediates the expression induction of more TGF-β target genes. One of these target genes is α-SMA. SMAD4 protein levels were affected by CM in a cell type-dependent manner (**Figures 5D–F**; **Additional File 2**). Both increased and decreased levels were observed. The levels of the TGF-β target gene α-SMA were significantly increased by both CM in the two UCC with epithelial morphology (**Figures 5G, H**; **Additional File 2**), but not in UMUC-3 cells that already display a mesenchymal phenotype. These results indicate that both CM can activate TGF-β signaling and that EMT may be induced in UCC with an epithelial phenotype, but not in UCC with a mesenchymal phenotype.

Since the loss of E-cadherin and the gain of vimentin protein levels are known indicators of EMT induction, we performed Western blot analysis (**Additional Files 2**, **3A–F**) and immunocytostaining for E-cadherin and vimentin protein (**Additional Files 3G–I**). However, even though we could not detect the loss of E-cadherin protein, vimentin was significantly increased by both CM in BFTC-905 cells. Surprisingly, vimentin levels were significantly decreased by both CM in epithelial type VMCUB-1 cells. Mesenchymal UMUC-3 cells did not display relevant changes.

### 3.3 Identification of secreted factors mediating the effects of CM

**Figure 6:**
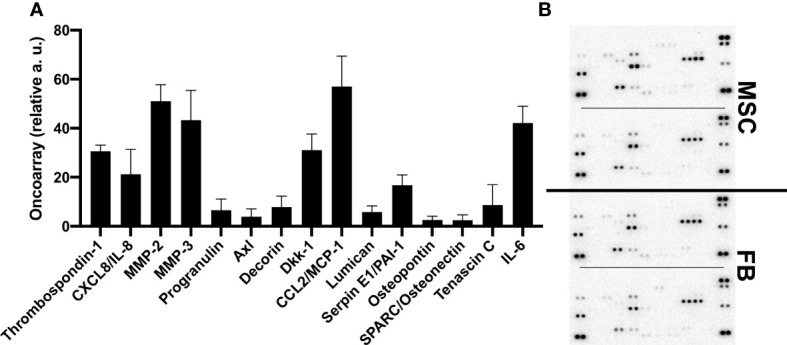
Quantification of proteins secreted by MSC and FB. Proteome arrays were performed with conditioned media from FB and MSC and applied to oncoarray membranes. Signal intensity was quantified by means of two for indicated proteins. Mean values across FB- and MSC-CM from different donors (*n* = 4) are displayed **(A)**. Raw data images of developed array membranes are displayed **(B)**.

To obtain more information about the cytokines and proteins contained in the CM that could stimulate the observed effects and molecular changes, oncoarray protein arrays were performed for 19 different proteins. Interestingly, the oncoarray results were very similar between FB-CM and MSC-CM, and data was merged (**Figures 6A, B**). Eight of the 19 analyzed proteins were robustly detectable, namely, thrombospondin-1, IL-8 (CXCL8), MMP-2, MMP-3, DKK-1, MCP-1/CCL2, Serpin E1/PAI-1, and IL-6. The function of identified proteins in general and with relation to bladder cancer is summarized in **Additional File 4**.

### 3.4 Identification of CM-induced molecular changes by next-generation RNA sequencing

Since we observed several significant changes in cellular properties towards a more aggressive phenotype in UCC by TME-conditioned media, we aimed at deciphering underlying signaling pathways and molecules in more detail than by protein analyses for selected proteins. To this end, we performed transcriptomic analysis by RNA sequencing. Since most prominent changes were observed in epithelial-like BFTC-905 and VMCUB-1 cells, both cell lines were used for RNA sequencing analysis after treatment with either MSC- or FB-CM and compared with DMEM controls. Overall, the numbers of significantly differentially expressed genes (DEG) were in the same range over different treatment conditions, with the exception of BFTC-905 which responded more strongly by altered gene expression to FB-CM than VM-CUB1 cells (**Table 1**).

**Table 1:** DEG numbers after treatment of BFTC-905 and VM-CUB1 cells with MSC-CM or FB-CM.

	BFTC-905 MSC	VM-CUB1 MSC	BFTC-905 FB	VM-CUB1 FB
Genes upregulated	1,159	1,255	1,721	1,236
Genes downregulated	1,320	1,155	1,869	1,069

Fold change ≥1.5 and Bonferroni adjusted p-value ≤0.05 were the cutoff values.

**Figure 7:**
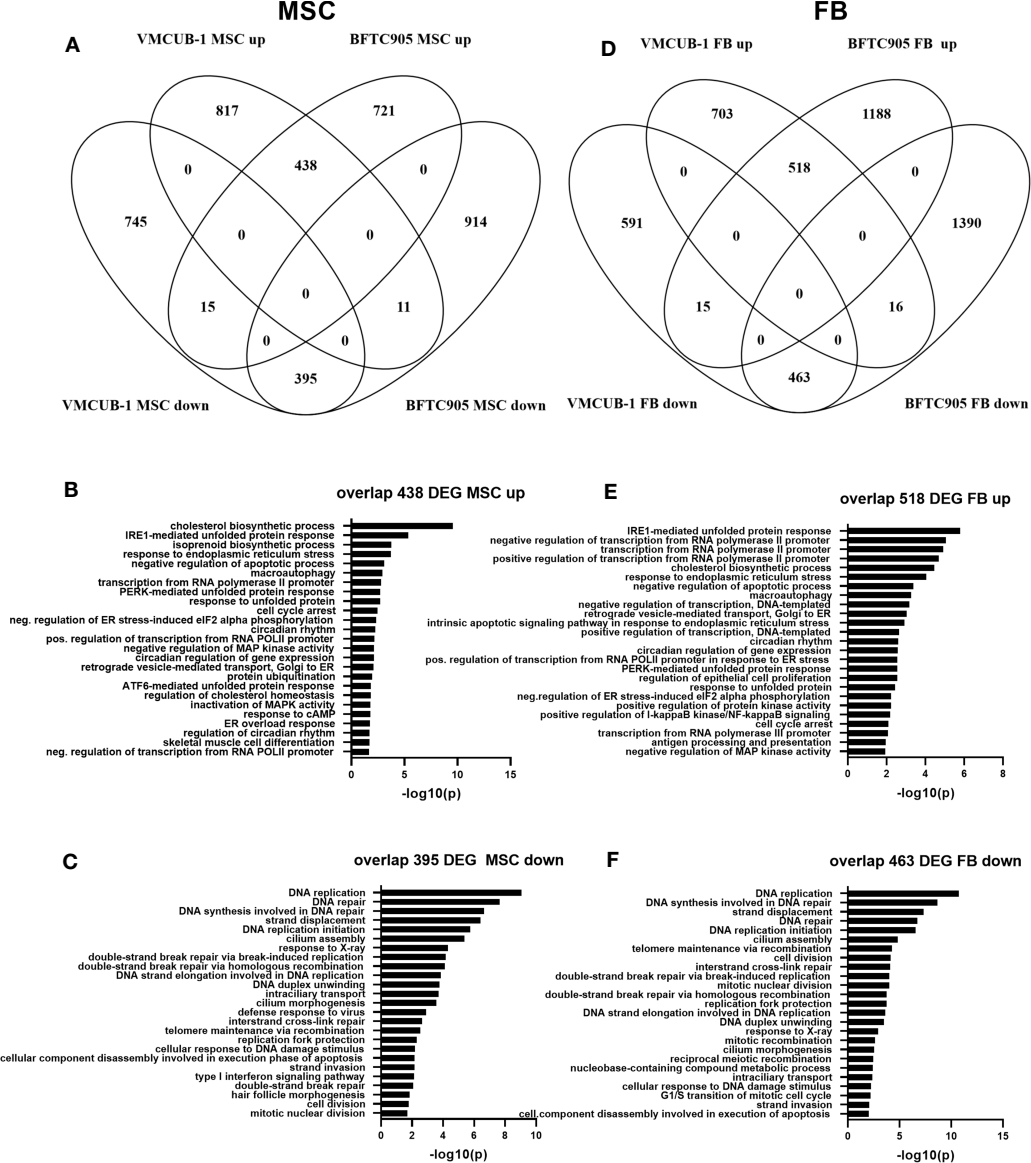
Transcriptome changes induced by conditioned media. RNA sequencing was performed to characterize transcriptomic changes induced by the indicated CM in BFTC-905 and VM-CUB1 cells. Significantly differentially expressed genes (fold change ≥1.5 and Bonferroni adjusted *p*-value ≤0.05) were compared by Venn diagrams between cell lines for MSC-CM **(A)** and FB-CM **(D)** using Venny 2.0 (52). The indicated numbers of genes in the overlap of MSC-CM upregulated **(B)** or downregulated **(C)** genes were subjected to gene GO analysis using DAVID online tool (58). FB-CM affected genes were likewise analyzed **(E, F)**.

In the first step of data analysis, we aimed to identify the cell line-independent effects of MSC-CM. To this end, we compared the MSC DEGs between BFTC-905 and VMCUB-1 cell lines (**Figure 7A**). Both cell lines shared 438 upregulated genes and 395 downregulated genes; only very few genes were oppositely expressed (**Additional File 5**). Common genes were subjected to gene ontology (GO) analysis to determine the cellular processes enriched for candidate genes. GOs were sorted by significance, and the TOP25 GOs for upregulated genes are displayed in **Figure 7B**. Metabolic processes like cholesterol and isoprenoid biosynthesis were among the GOs related to shared upregulated genes. This may be related to the origin of MSC cultures derived from adipose tissue and their dependency on cholesterol biosynthesis for proliferation. According induced metabolic reprogramming in UC cells may add to growth stimulation (43, 59). Furthermore, the regulation of transcription, cellular response to unfolded protein, and endoplasmatic reticulum (ER) stress were affected. A significant number of genes were also enriched in negative regulation of apoptosis.

The gene expression changes of selected genes were further validated by qRT-PCR. The expression of anti-apoptotic regulators, namely, the Bcl-family members Bcl-2 and Bcl-XL, was slightly altered in a cell line-dependent manner. The survivin levels were not significantly altered (**Additional File 6**). Examples of strongly induced genes by MSC-CM contributing to enhanced aggressive properties, particularly migration, invasion, and cisplatin resistance of both BFTC-905 and VMCUB-1 were *Snail 1* (*SNAI1*) and *SQSTM1* encoding for p62 protein involved in the reduction of cisplatin which reduced cell stress *via* NRF-2 signaling-mediated detoxification and activation of NF-κB signaling (**Additional File 7A**). DEGs associated with inflammatory response like *TNFα*, as well as molecules involved in antitumor defense by immune cells like *MICB*, were also identified and validated by qRT-PCR (**Additional File 7B**).

The TOP25 GOs of common MSC downregulated DEGs were dominated by various processes of DNA damage response, DNA repair, and cell cycle. Homologous recombination (HR) repair genes like *BRCA1/2*, *XRCC2*, or *RAD51* were downregulated by MSC-CM (**Figure 7C**; **Additional File 7C**). Type I IFN signaling and response to virus were also among the GOs of downregulated GOs, suggesting that cGAS-STING signaling, also known as viral mimicry, may be negatively affected by MSC-CM. Examples for downregulated genes were *MX1*, *OASL*, *OAS2*, and *IFIT2*. Some effects of MSC-CM were cell line dependent.

Our ICC and Western blot results for E-cadherin and vimentin protein changes indicated the induction of EMT in MSC-treated BFTC-905 cells, but not in VMCUB-1 cells. Concurringly, the qRT-PCR validation of *SNAI1*, *TWIST1*, and *CLDN4* also demonstrated stronger expression changes in BFTC-905 cells (**Additional File 7A**). Likewise, *CK14*, a marker for less differentiated cells with epithelial plasticity, and *DKK1*, promoting proliferation and invasion, were strongly induced in BFTC-905 cells, but not in VMCUB-1 cells.

DEGs that were unique for either BFTC-905 or VMCUB-1 cells were also subjected to GO analysis (**Additional Files 8**, **9A–E**, **10**). A strongly increased expression of *Cyclin A*, *D*, and *E* in BFTC-905 cells may contribute to their enormously increased cell proliferation (**Additional File 8D**) that was observed compared to cells treated by FB-CM and compared to VMCUB-1 cells. Histone acetylation was unique among the downregulated DEGs in BFTC-905 (**Additional File 8E**).

Concurring with the observed strong stimulatory effect of MSC on the migration capacity of VMCUB-1 cells, the upregulated DEGs in VMCUB-1 cells were enriched in actin filament-based movement, microtubule organization, and cell polarity (**Additional File 9D**). Metabolic processes and *IDO1*-modulating T cell behavior were downregulated in VMCUB-1 cells (**Additional Files 7B**, **9E**).

Next, we analyzed FB-CM-induced gene expression changes likewise. Both cell lines shared 518 upregulated genes and 463 downregulated genes; again, only very few genes were oppositely expressed (**Figure 7D**). Since BFTC-905 cells responded more strongly to FB CM, the number of unique BFTC-905-regulated genes was higher (**Table 1**; **Additional Files 5**, **8A**).

The TOP25 GOs of upregulated DEGs shared by both cell lines overlapped with MSC-affected GOs. The GOs were sorted by significance. The TOP25 GOs are displayed in **Figures 7E, F**. Again, cholesterol biosynthesis, regulation of transcription, cellular response to unfolded protein, ER stress, and negative regulation of apoptosis were affected. Altered cholesterol lipoprotein metabolism in cancer cells may contribute to growth advantage (60, 61). Also, macroautophagy was enriched. Furthermore, GOs affected by FB-CM in **Figure 7E** show a negative regulation of MAP kinase activity, a positive regulation of NF-κB signaling, and regulation of cell proliferation. However, these GOs were also among MSC-affected GOs, but not among the TOP25 and thus not displayed in **Figure 7B**.

The qRT-PCR validation of the above-mentioned gene expression changes revealed that *CLDN4* was induced by FB media in both cell lines much more strongly than by MSC media (**Additional File 7A**). Also, *SQSTM1* and TNFα were more strongly induced by FB-CM than MSC-CM, concurring with finding the activation of NF-κB signaling among TOP25 FB GOs. Again, *MICB* was also strongly induced by FB-CM (**Additional File 7B**).

The TOP25 FB GOs of downregulated DEGs were even more dominated by various processes of DNA damage response, DNA repair, and cell division compared to MSC-treated UCC (**Figure 7F**). FB-CM highly induced DNA damage response marker *GADD45B* in both cell lines (**Additional File 7C**). Response to virus (cGAS-Sting) was also affected, though not among TOP25; in contrast to the MSC effect, type I IFN signaling was not altered by FB-CM according to GO analysis. However, individual genes like *MX1* were downregulated (**Additional File 7B**).

Some cell line-dependent effects of FB-CM were comparable with those of MSC-CM. Again, *SNAI1* was rather induced in BFTC-905 cells than in VMCUB-1 cells, as were *CK14* and *DKK1* (**Additional File 7A**). Obviously, FB-CM can also contribute to the more aggressive properties of UCC and increase the epithelial plasticity in BFTC-905 cells.

Likewise, *CXCL10* was instead induced in BFTC-905 cells. In BFTC-905, several genes associated with cisplatin resistance were strikingly induced by FB-CM, but not by MSC-CM. The induced genes were nuclear factor erythroid 2-related factor 2 (*NRF-2*), glutathione peroxidase (*GPX*) 1, *GPX2*, and saliva level of solute carrier family 3 member 2 (*SLC3A2*), which are all involved in detoxification.

FB DEGs that were unique for the investigated cell lines were also subjected to GO analysis (**Additional Files 8**–**10**). While ribosomal biogenesis was enriched among upregulated DEGs unique for FB-CM-treated BFTC-905 cells (**Additional File 8B**), various immune/inflammatory responses were enriched among upregulated DEGs unique for FB-CM-treated VMCUB-1 cells (**Additional File 9B**). Cell line-dependent FB-CM downregulated genes enriched in transcriptional regulation in BFTC-905 cells (**Additional File 8C**) and regulation of cell cycle and cell division in VMCUB-1 cells (**Additional File 9C**).

### 3.5 Differences in gene expression alterations between MSC- and FB-CM-treated UCC

Taken together, treatment with both MSC- or FB-CM often induced similar changes in cellular processes in UCC. Concurringly, 954 and 810 DEGs in VMCUB-1 cells overlapped between FB- and MSC-regulated genes (**Additional File 9A**) and 1,126 and 1,161 DEGs were in the overlap of BFTC-905 cells (**Additional File 8A**). While in BFTC-905 the number of genes uniquely affected by MSC-CM was very small compared to uniquely FB-CM regulated genes, the opposite was observed for VMCUB-1 cells, suggesting differences in the responsiveness of UCC to different cell types of TME. To identify those processes uniquely affected by either FB or MSC in a cell line-dependent manner, the respective unique DEGs were subjected to GO analysis.

The unique FB effects in BFTC-905 cells were related to chromatin remodeling enzymes, zinc finger nucleases, regulation of translation and protein folding, vesicle formation and transport (Golgi, autophagy), and response to reactive oxygen species (**Additional Files 8A–C**).

Unique MSC effects in BFTC-905 cells involved interstrand crosslink repair and factors involved in histone and protein acetylation, but not classic histone acetyltransferase or deacetylases. Upregulated genes regulated the blood vessel morphogenesis and sprouting angiogenesis (**Additional Files 8D, E**).

Unique FB effects in VMCUB-1 cells were involved in antigen presentation, immune response, IFN γ signaling, and T cell interaction. Downregulated genes were frequently involved in DNA replication, repair, and chromosome segregation (**Additional Files 9A–C**).

The unique MSC effects in VMCUB-1 cells were various metabolic processes like cholesterol, insulin, and lipid metabolism as well as microtubule organization, polarity, and focal adhesion (**Additional Files 9D, E**).

## 4 Discussion

Treatment resistance, e.g., to cisplatin-based chemotherapy is a major limitation of therapy not only for UC patients (62–64). Cisplatin-resistant cancer cells utilize a plethora of resistance mechanisms that differ even between tumor cells within one cancer entity (65). Thus, therapy resistance cannot be overcome by targeting one individual signaling pathway; a broader treatment approach needs to be developed instead.

Lately, there is growing evidence that cellular components in the TME, like MSC and FB, can also affect cancer development and chemoresistance (12, 23, 35, 66–68). However, the impact of such cells can be either tumor-promoting or tumor-inhibiting (69) and can be tissue-dependent as demonstrated by a meta-analysis of data on the effect of MSC-derived CM on various human cancer cell lines (70). This analysis of 47 publications included only one bladder cancer cell line. The T24 cell line was reported by Maj et al. to respond with inhibited cell proliferation to MSC-CM (71). Only few further studies on human bladder cancer cell lines 5637 and HT-1376 have been performed since then, with partially inconsistent results for 5637 (72, 73). Differing results for one cancer entity or even the same cell line may originate from the heterogeneity of MSC cultures. It is well known that their properties and effect depend on their source (e.g., bone marrow- or adipose-derived), characteristics of specific donors (like age), or the passage number of MSC cultures (47, 74, 75).

Accordingly, further investigations on the effect of TME in bladder cancer, particularly for poorly investigated MSC, are needed because TME affects the relevant properties of cancer cells and, importantly, the patients’ response to the standard-of-care treatment. If TME cells survive standard chemotherapy, they may contribute to tumor relapse by promoting the outgrowth of chemoresistant tumor clones from residual disease (76, 77). Once the functional role of TME cells in urothelial cancer is understood, innovative treatment regimens targeting TME cells can be developed.

Thus, the primary aim of our study was to characterize the effects of CM from TME cells on UC cells in detail. We decided to use FB and MSC. FB are a known major TME component and were well investigated in the past [recently reviewed in (11)] so that we aimed to used them in comparison to the less well-investigated MSC. MSC also have the capacity to differentiate into various tumor-associated cells, also activated FB (CAFs) (78). Furthermore, for other cancer entities, their relevance for treatment response has been reported, e.g., for colon cancer (42) and prostate cancer (79). To prevent inconsistencies originating from heterogenic primary cultures, we followed a more standardized approach. We used defined cell numbers of proliferating primary cell cultures in the early passage (only passage numbers 3–8) of six different donors to manufacture one pooled CM. We subsequently utilized this as one “n”. Since UC is a heterogenic disease, we selected three different UC cell lines, two with an epithelial phenotype and one with a mesenchymal phenotype.

Generally, we observed that the CM of MSC and FB had consistent growth-promoting effects in all three UC cell lines. However, the impact of MSC-CM was much stronger and the effect was most prominent in BFTC-905 and UMUC-3 cells compared to VMCUB-1 cells. The proliferation-promoting effects of MSC on the cancer cells of other entities, e.g., ovarian, colorectal, and liver cancer, were already reported (80–82). MSC are thought to reprogram the energy metabolism of cancer cells and to support the latter with high-energy metabolites (43), which may explain why we observed stronger growth-promoting effects by MSC-CM than FB-CM. Our observed differences in the proliferation activity between UCC were also reflected at the molecular level in our RNA and protein analyses. MSC and FB induced more CK14 and DKK1 in BFTC-905 than in VMCUB-1 and thus could be jointly responsible for the increased proliferation and more epithelial plasticity, also contributing to chemoresistance (83, 84). Likewise, the expression of cyclins and CXCR-4 was most strongly induced in BFTC-905 cells by MSC-CM compared to VMCUB-1. Several working groups reported that CXCR-4 mediates proliferation and migration *via* MAPK or PI3K/Akt pathways (85) and that CXCR-4 is involved in the regulation of Cyclin D1 expression (86). CXCR-4 has been identified as a potential target for breast cancer treatment. Tripathy et al. could demonstrate that blocking CXCR-4 with a specific antibody (AMD3100) synergizes with docetaxel and led to abnormal mitosis in resistant cell lines (87). Thus, our results may indicate that AMD3100 could also be suitable for synergistic therapy in UC.

Concurringly, more broad GO analyses of our RNA Seq results confirmed a strong impact of MSC-CM on proliferation in BFTC-905 cells. At the protein level, we found very few differences between MSC- and FB-CM in secreted proteins by oncoarray analyses, which demands whole secretome analyses in the future. MSC-secreted factors known to activate tumor cell growth and proliferation of other cancer entities are, for example, CCL2/MCP-1, angiogenin, and VEGF (44, 88, 89). We could show that high levels of secreted CCL2 and IL-6 were detectable in both FB- and MSC-CM, which could also be targeted in the future.

When we additionally treated UC cells with cisplatin, we found that the CM of both TME cell types reduced the cisplatin sensitivity of all three UCC. Thus, to our knowledge, we demonstrate for the first time that MSC do affect the response of bladder cancer cells to chemotherapy with cisplatin. Earlier studies on UC reported similar affects regarding treatment with ciprofloxacin, an antibiotic given in the case of urogenital infections (71).

To analyze whether UC cells acquire a more aggressive phenotype by treatment with CM, we investigated migratory and invasive capacity and molecular markers. While MSC-CM significantly increased the migration capacity of all three investigated UC lines, FB-CM did not. Both CM types strongly increased the invasive potential.

Concurringly, the GO analysis of RNA Seq data revealed an upregulation of smooth muscle contraction, with cell migration involved in sprouting angiogenesis in BFTC-905 cells. Response to mechanical stimulus, actin filament-based movement, establishment of monopolar cell polarity, and cytoplasmatic microtubule organization were induced in VMCUB-1 cells. To our knowledge, our study provides the first RNA Seq data set from functional experiments with different UCC treated with conditioned media of MSC compared to FB. Concurringly, data on the impact of MSC on bladder cancer cells is scarce; however, MSC-induced amplification of aggressive phenotypes was already observed in cancer cells from lung and breast and in melanoma (66, 90, 91). Melanoma cell behavior was influenced by TGF-β (91), and the invasive capacity of breast cancer cells was modified by MSC-secreted CCL5 and CCL9 and the activation of MMP (92) as well as by the induction of EMT *via* activated ERK signaling (93).

Also, data on the impact of FB on bladder cancer cells is limited. Early immunohistochemical analyses of UC patient tissues demonstrated that the abundance of FB subpopulations correlated with EMT and tumor progression (94). One report on bladder cancer cell lines (5637, T24, J82, HT1376, and MGHU-1 cells) demonstrated that CM from FB increased the invasion capacity and identified secreted HGF as one mediating soluble factor (95). FB effect is better studied in other cancer entities, particularly in breast cancer. It is well known that FB modulate the TME supporting tumor expansion and invasion. Apart from FB-secreted pro-inflammatory cytokines and chemokines, another promoting factor described is tissue polysaccharide hyaluronan (HA) through binding to HA receptors like CD44 and RHAMM/HMMR. In breast cancer, increased HA receptor expression is even prognostic for poor outcome disease recurrence (96). We also observed increased CD44 levels for UC lines with epithelia morphology after treatment with CM. CD44 could be a promising tool to address the TME and improve bladder cancer therapy as already demonstrated for pancreatic ductal adenocarcinoma. A doubled overall survival could be demonstrated there using a combination of chemotherapeutic regimen and pegvorhyaluronidase *α* (PEGPH20), an enzymatic agent that can rapidly reduce the HA level (97, 98). In addition, Hoffmann and colleagues published earlier that CD44v6, an isoform of CD44, is a suitable target for the targeting of CAR T cells (99).

In the literature, FB were also reported to induce EMT in breast cancer cells by the secretion of activating growth factors like TGF-β1, EGF, PDGF, HGF, and MMP (100). Our analysis of the selected secreted factors in CM by oncoarrays also proved the secretion of chemokines like CCL2, IL6, and IL8. The only earlier published results of MSC effects on 5637 and HT-1376 bladder cancer cell lines by ELISA reported cell line-dependent effects on IL-1B, IL-6, IL-8, TNFα, GM-CSF, MCP-1, TGF-β, and RANTES (72). Thus, with our results, new treatment options for UC targeting the interaction between UC and TME cells are emerging, as it has already been demonstrated that small-molecule inhibitors targeting CCL-2 as Bindarit inhibit tumor progression and metastasis in a breast cancer cell line and in prostate cancer xenograft mice (101).

Furthermore, we found the MMP-2 and MMP-3 levels to be increased in CM. PAI-1 and SMAD4 were further analyzed to prove the activation of TGF-β signaling by CM treatment. After TGF-β signaling activation, the TGF-β receptor phosphorylates SMAD2 and SMAD3 proteins that complex with SMAD4, translocating to the nucleus where EMT-related genes as E-cadherin, PAI-1, and *α*-SMA will be regulated (102). However, PAI-1 is not only associated with EMT but additionally promotes cell invasion and migration (103), accelerates cell proliferation, and protects the cell from apoptosis (104). In bladder cancer patients, PAI-1 expression correlated with tumor grade, tumor stage, and overall patient survival (105).

Since TGF-β can induce EMT (106), we also determined the levels of EMT markers. Downregulation of E-cadherin and upregulation of vimentin and α-SMA are well known characteristics of EMT (107). Our protein analyses for E-cadherin and vimentin did not result in a clear result, maybe due to only partial EMT induction. It is well known that EMT may be a continuum with intermediate states. However, α-SMA was clearly induced in the two cell lines with an epithelial phenotype. In addition, the RT-PCR validation of RNA Seq candidates demonstrated that the expression of EMT regulators like *SNAI1*, *TWIST1*, and *CLDN4* (108–113) was induced by both CM, proving the induction of EMT in BFTC-905 and VMCUB-1 cells.

It is well known that, also in bladder cancer, EMT is associated with invasion and metastasis as well as drug resistance (114–116). Concurringly, we observed that FB- and MSC-CM significantly accelerated cisplatin resistance. In a previous work, we extensively studied molecular cisplatin resistance mechanisms that are intrinsically activated in UC cells and act on different levels of cisplatin detoxification (65, 117, 118). These aforementioned mechanisms also seem to be activated by CM treatment. We reported that cisplatin-resistant UC cells gain an induced capacity of detoxification by the deregulated expression of ROS detoxification molecules (117). Key factors mediating ROS detoxification are NRF-2 and NRF-2 target genes (*GPX1*, *GPX2*, and *SLC3A2*) regulating drug uptake/efflux and antioxidant response *via* glutathione metabolism (119, 120). Concurringly, for example, BFTC-905 cells treated with FB-CM displayed elevated levels of *NRF-2*, *GPX1*, *GPX2*, and *SLC3A2* which may lead to the observed increased cisplatin resistance. Furthermore, CM-treated BFTC-905 and VMCUB-1 cells had p62 and TNF*α* upregulated. Also, p62 is involved in cisplatin detoxification *via* NRF-2 signaling and activation of NF-κB signaling. The latter signaling cascade can also be activated by TNF*α*, which, in turn, is associated with apoptosis prevention, upregulation of EMT markers, tumor progression, and cisplatin-induced chemoresistance in bladder cancer (121).

Also, interferon (IFN) signaling may be involved in the mediation of chemoresistance. Intact IFN type I signaling impacts on the efficacy of cytotoxic drug therapy, radiotherapy, and targeted immunotherapies and depends on both direct tumor cell inhibition and indirect anti-tumor immune response. Thus, malfunctions of IFN signaling, as observed in hypoxic TME or in immune cells, may be a causative factor behind the therapeutic resistance in cancer patients (122, 123). Interestingly, MSC-CM negatively affected IFN type I signaling and IFN-inducible antiviral effectors such as *OAS2*, *OASL*, *MX1*, and *IFIT2*.

Interferon genes may also be related to DNA damage *via* the cyclic GMP-AMP synthase (cGAS)-STING pathway. cGAS senses cytosolic double-stranded DNA (dsDNA), resulting in the synthesis of cyclic GMP-AMP, a secondary messenger binding to the adaptor protein STING. It is known that TME can impact the DNA repair pathways in cancer cells (124). Once cisplatin–DNA adducts have been formed and DNA is damaged, cisplatin tolerance could be mediated by different DNA repair mechanisms, such as nucleotide excision repair (NER), base excision repair (BER) (112), or repair of DNA double-strand breaks (DSB) by non-homologous end joining (NHEJ) or homologous recombination (HR). The known mechanism how TME may regulate DNA repair capacity is the cGAS interaction with PARP, inhibiting HR through the downregulation of repair molecules like RAD51 and RPA2. DNA repair factors have also been shown to be reduced by hypoxia induced by TME (124). Concurringly, we found DNA repair molecules like BRCA2 and RAD51 to be reduced by CM, and the DNA damage response marker GADD45B increased. Theoretically, reduced DNA repair capacity rather argues against increased chemoresistance. However, common sense in the literature is that the TME may impair the DNA repair of highly proliferating cancer cells, leading to the accumulation of further DNA damage and increased genetic instability. Increased genetic instability and high mutational burden may then secondarily contribute to chemoresistance (67). Of note is that muscle-invasive bladder cancer is one of the most genetically instable cancer entities anyway.

In conclusion, our analyses clearly demonstrated consistently that conditioned media from MSC and FB affected various properties of UC cells, resulting in a more aggressive phenotype. Concurring with data from literature on other cancer entities, we confirmed for the first time that these candidate factors mediate the observed effects also in bladder cancer cells. Such factors could also be putative targets for future therapeutic approaches targeting the interaction between TME and bladder cancer cells to overcome treatment resistance or prevent the disease relapse of UC patients. For a deeper understanding of the underlying mechanism in the TME of bladder cancer, direct co-culture models with the above-mentioned new combination treatment approaches, high throughput secretome analyses, and validation studies in patient tissues should be performed hereafter.

## Data availability statement

The original contributions presented in the study are publicly available at NCBI GEO. This data can be found here: https://www.ncbi.nlm.nih.gov/geo/query/acc.cgi?acc=GSE242217.

## Ethics statement

The studies involving humans were approved by the Department of Plastic Surgery, Hand Surgery, Burn Center, Merheim Hospital Cologne, University of Witten/Herdecke, Köln Köln (study no. 78/2017 approved by the local Ethics Committee June 08, 2017). The studies were conducted in accordance with the local legislation and institutional requirements. The participants provided their written informed consent to participate in this study.

## Author contributions

Conception and design: VG and MH. Administrative support: JW and BB. Provision of study materials or patients: VG and MH. Collection and assembly of data: LF, BF, KK, and PP. Data analysis and interpretation: LF, BF, KK, PP, VG, and MH. Manuscript writing: VG and MH. All authors contributed to the article and approved the submitted version.

## Supplementary material

Additional File 1 Primer sequences used for qRT-PCR validation.

Additional File 2 Protein analysis of CXCR4, SMAD4, *α*-SMA, E-cadherin, and vimentin. The raw Western blot images used for quantification in **Figures 4**, **5** and **Additional File 3** are displayed. For better comparability, we compared the abundance of target proteins to total protein using the stain-free technique. The advantage of the stain-free method is improved precision compared to normalization to actin and β-tubulin.

Additional File 3 Protein analysis of E-cadherin and vimentin. Western blot analysis of the protein levels of E-cadherin **(A–C)** and vimentin **(D–F)** of CM-treated and untreated cells (treatment duration was 6 days). Protein levels were quantified and normalized to DMEM-treated controls. Bars represent mean ± SD of the individual experiments indicated (*n* = 3), **p* ≤ 0.05, ***p* ≤ 0.01, ****p* ≤ 0.001. **(G–I)** Immunofluorescence staining of the indicated UCC treated with MSC-CM or DMEM for E-cadherin (red), vimentin (green), and DAPI (blue). A merged image of all fluorescence channels was created.

Additional File 4 Function of secreted proteins identified by oncoarray in general and with relation to bladder cancer.

Additional File 5 Lists of common DEGs corresponding with the analyses displayed in .

Additional File 6 qRT-PCR analysis of anti-apoptotic regulators. Gene expression changes in UCC were measured by quantitative RT-PCR. Tata-box binding protein (*TBP*) was used as a reference gene. Gene expression changes in cells treated with indicated CM were normalized to the respective DMEM controls.

Additional File 7 qRT-PCR validation of gene expression changes identified by RNA sequencing. Gene expression changes in BFTC-905 and VMCUB-1 cells were validated by quantitative RT-PCR. Tata-box binding protein (*TBP*) was used as a reference gene. Gene expression changes in cells treated with indicated CM were normalized to the respective DMEM controls. Different groups of genes associated with plasticity **(A)**, immune response **(B)**, and DNA damage and repair **(C)** were investigated.

Additional File 8 Identification of DEGs unique for either MSC-CM or FB-CM in BFCT-905 cells. Unique genes for the respective treatment group were identified by Venn diagram **(A)** and subjected to GO analysis. Uniquely affected DEGs by FB-CM **(B, C)** and by MSC-CM **(D, E)** are displayed.

Additional File 9 Identification of DEGs unique for either MSC-CM or FB-CM in VMCUB-1. Unique genes for the respective treatment group were identified by Venn diagram **(A)** and subjected to GO analysis. Uniquely affected DEGs by FB-CM **(B, C)** and by MSC-CM **(D, E)** are displayed.

Additional File 10 Lists of unique DEGs corresponding with analyses displayed in **Additional Files 8**, **9**.
