# Supplementary figures and images for: Tumorigenic effects of human mesenchymal stromal cells and fibroblasts on bladder cancer cells
Source: Front Oncol. 2023 Sep 13;13:1228185. doi: 10.3389/fonc.2023.1228185 (PMC10534007; doi:10.3389/fonc.2023.1228185)

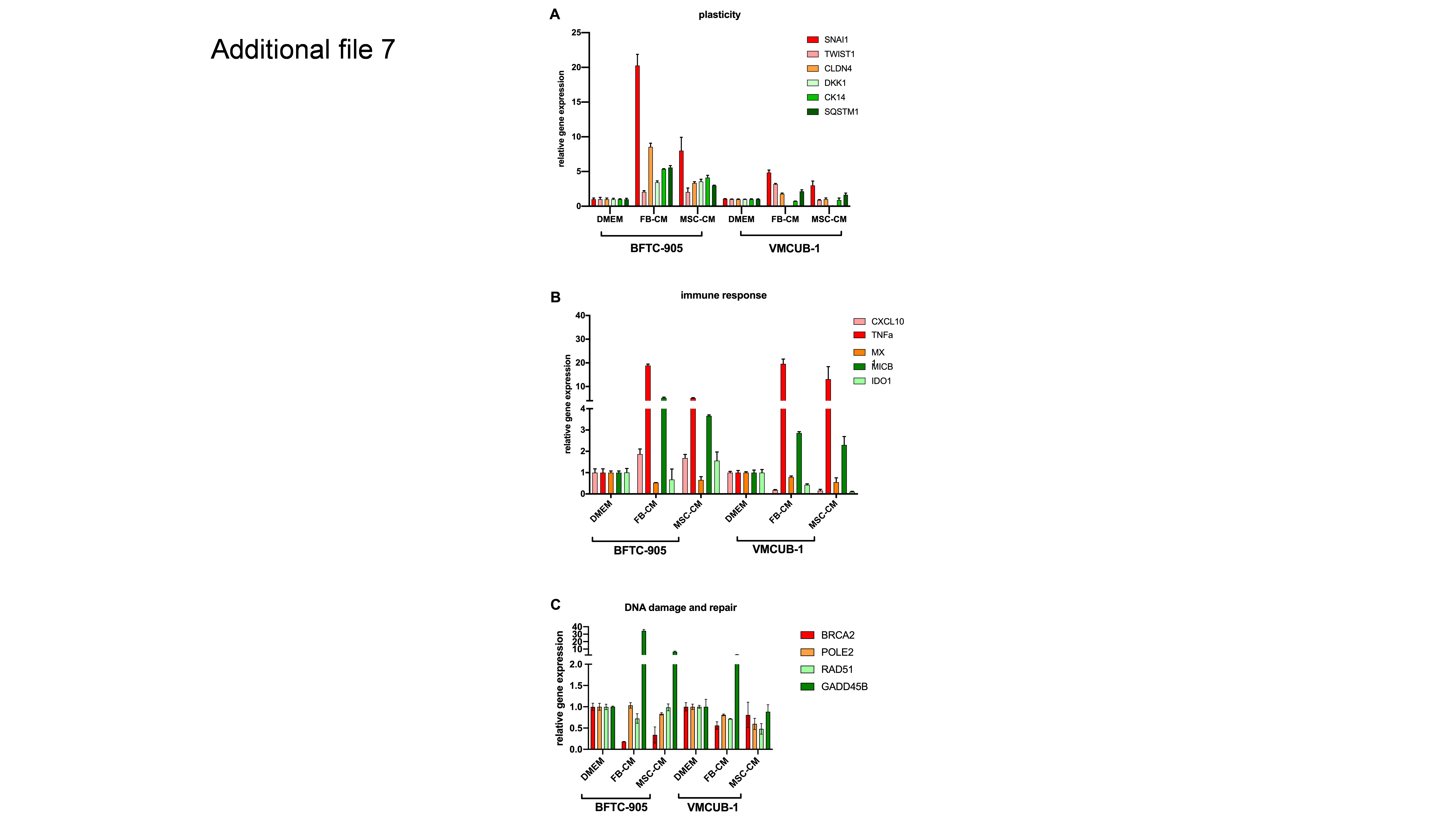

Supplement: Additional File 1 — Primer sequences used for qRT-PCR validation. [file Image_1.tif]

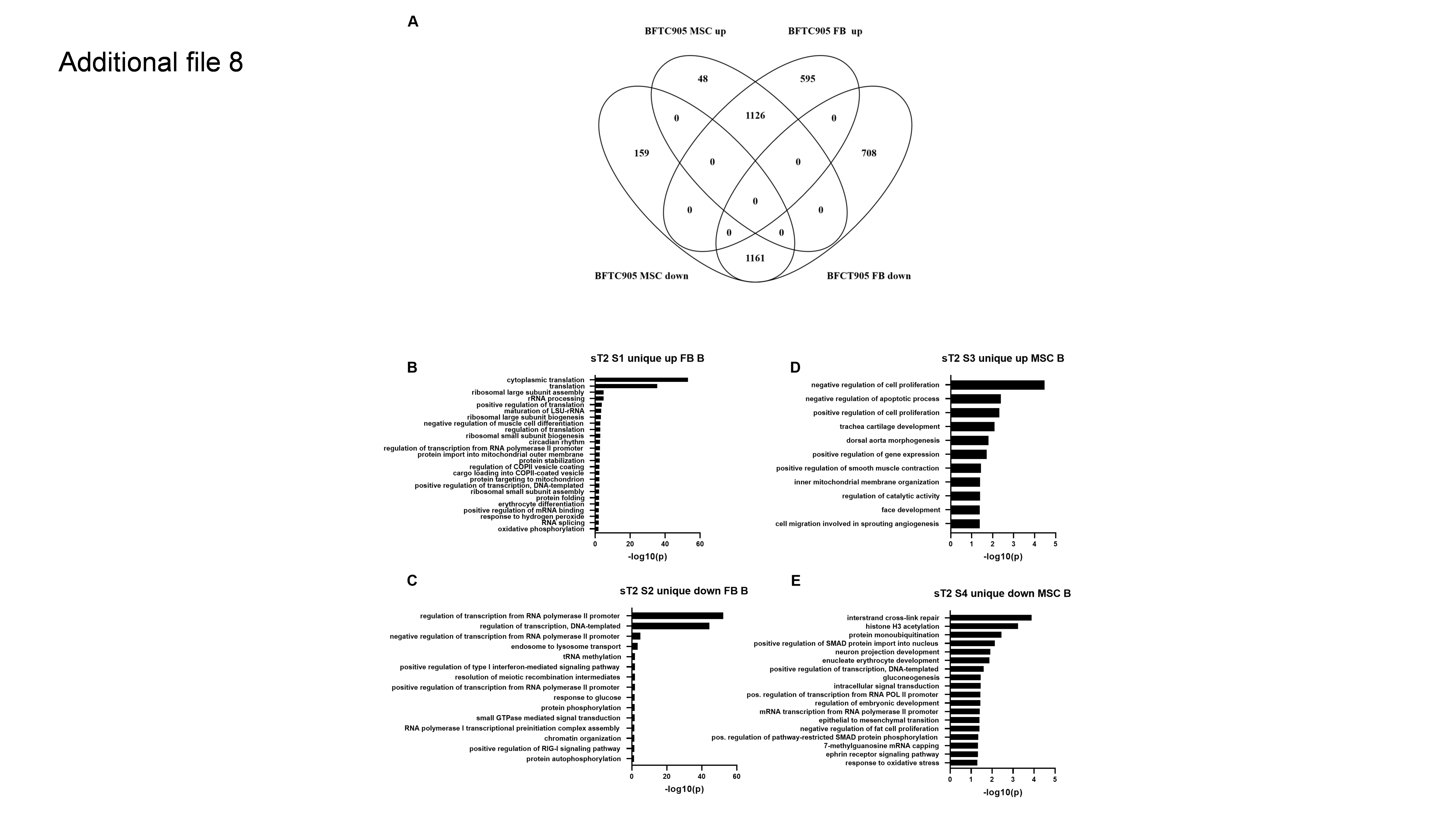

Supplement: Additional File 2 — Protein analysis of CXCR4, SMAD4, α-SMA, E-cadherin, and vimentin. The raw Western blot images used for quantification in Figures 4 , 5 and Additional File 3 are displayed. For better comparability, we compared the abundance of target proteins to total protein using the stain-free technique. The advantage of the stain-free method is improved precision compared to normalization to actin and β-tubulin. [file Image_2.tif]

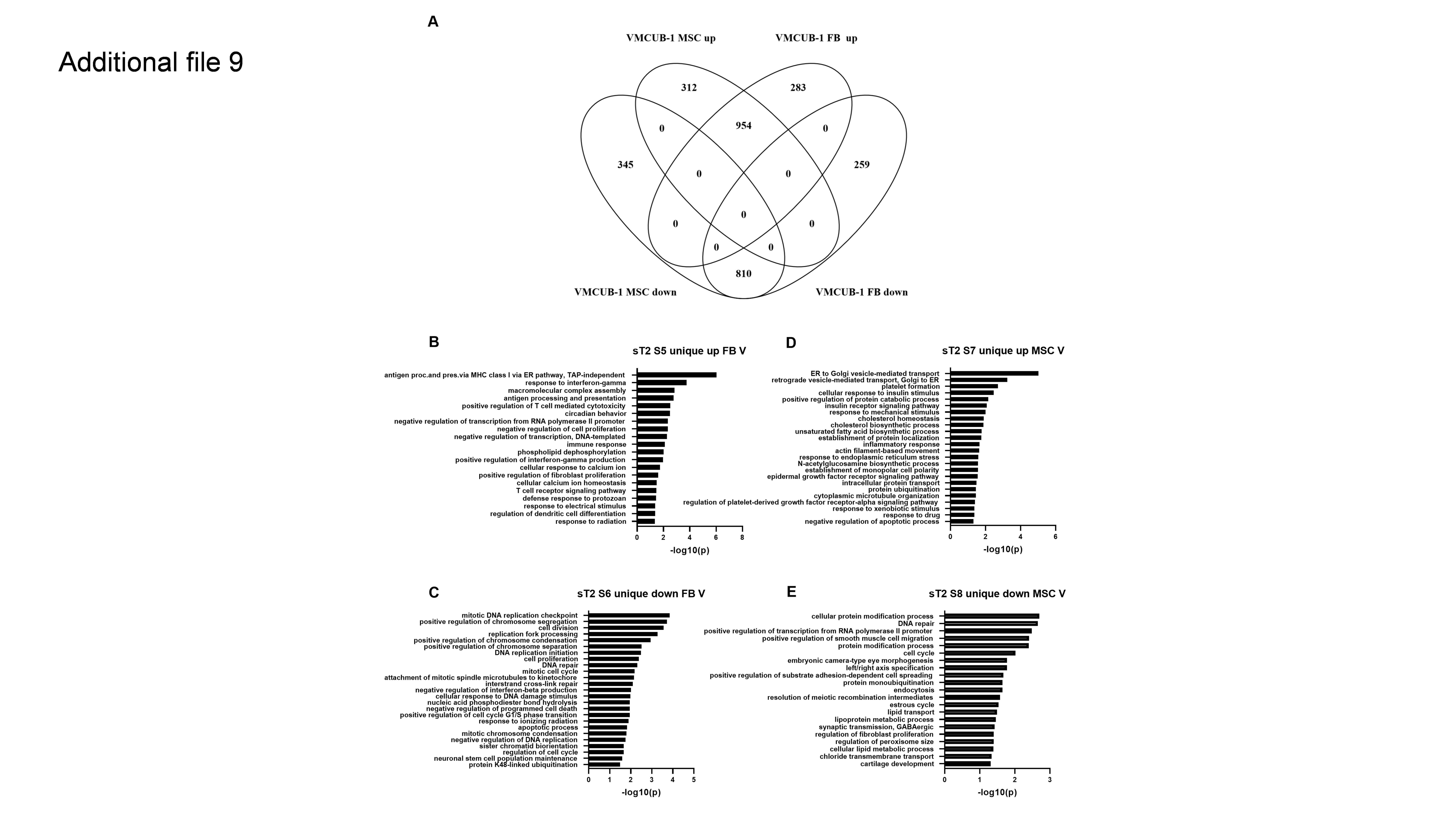

Supplement: Additional File 3 — Protein analysis of E-cadherin and vimentin. Western blot analysis of the protein levels of E-cadherin (A–C) and vimentin (D–F) of CM-treated and untreated cells (treatment duration was 6 days). Protein levels were quantified and normalized to DMEM-treated controls. Bars represent mean ± SD of the individual experiments indicated (n = 3), *p ≤ 0.05, **p ≤ 0.01, ***p ≤ 0.001. (G–I) Immunofluorescence staining of the indicated UCC treated with MSC-CM or DMEM for E-cadherin (red), vimentin (green), and DAPI (blue). A merged image of all fluorescence channels was created. [file Image_3.tif]

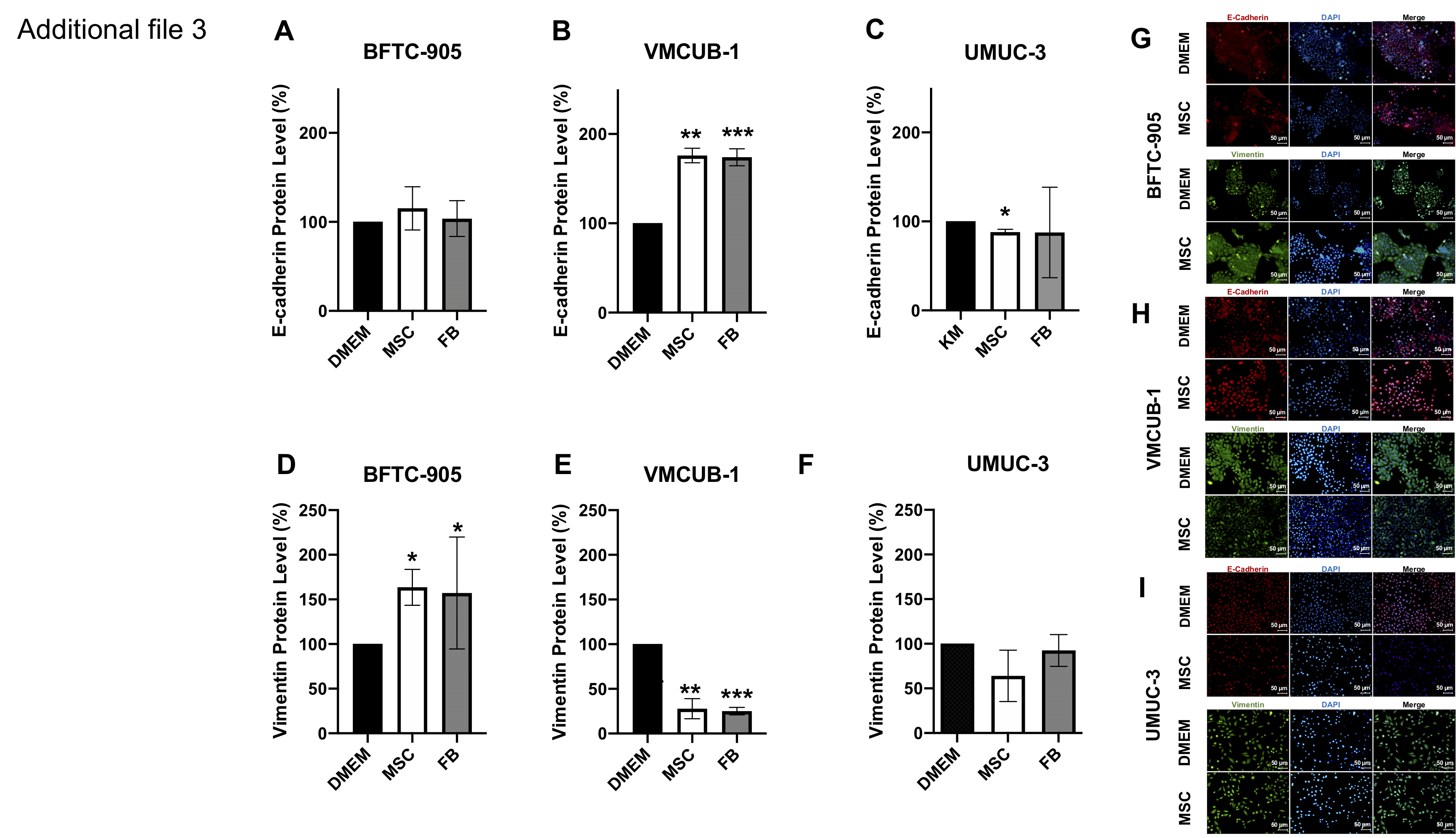

Supplement: Additional File 4 — Function of secreted proteins identified by oncoarray in general and with relation to bladder cancer. [file Image_4.tiff]

# Additional file 6

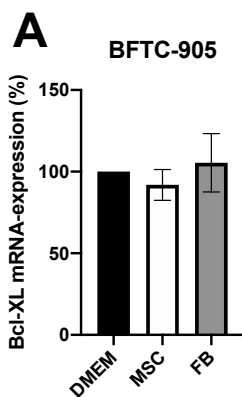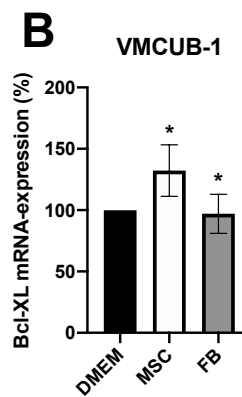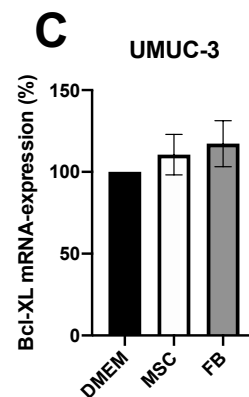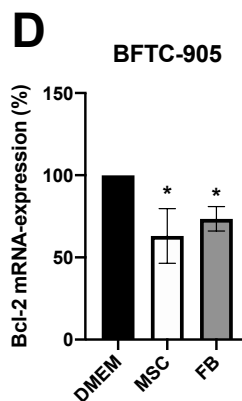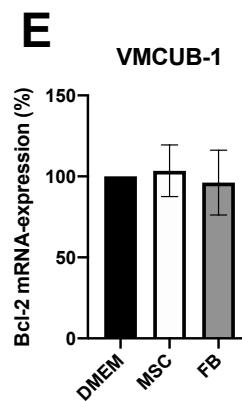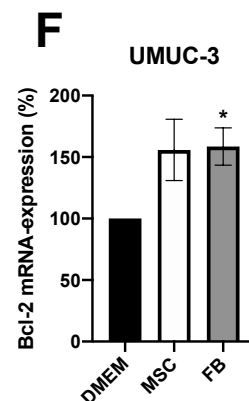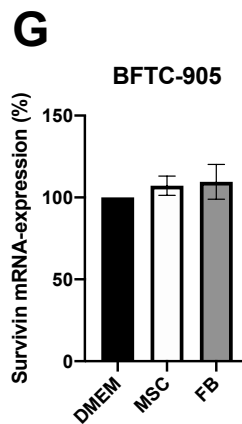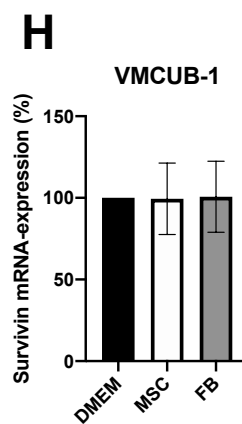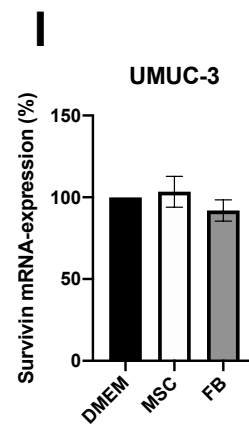

Supplement: Additional File 7 — qRT-PCR validation of gene expression changes identified by RNA sequencing. Gene expression changes in BFTC-905 and VMCUB-1 cells were validated by quantitative RT-PCR. Tata-box binding protein (TBP) was used as a reference gene. Gene expression changes in cells treated with indicated CM were normalized to the respective DMEM controls. Different groups of genes associated with plasticity (A), immune response (B), and DNA damage and repair (C) were investigated. [file DataSheet_3.pdf]
