# Supplementary material for: Tumorigenic effects of human mesenchymal stromal cells and fibroblasts on bladder cancer cells
Source: Front Oncol. 2023 Sep 13;13:1228185. doi: 10.3389/fonc.2023.1228185 (PMC10534007; doi:10.3389/fonc.2023.1228185)

Additional file 2

Western Blot analyses using Stain-Free technique

CXCR-4 BFTC-905

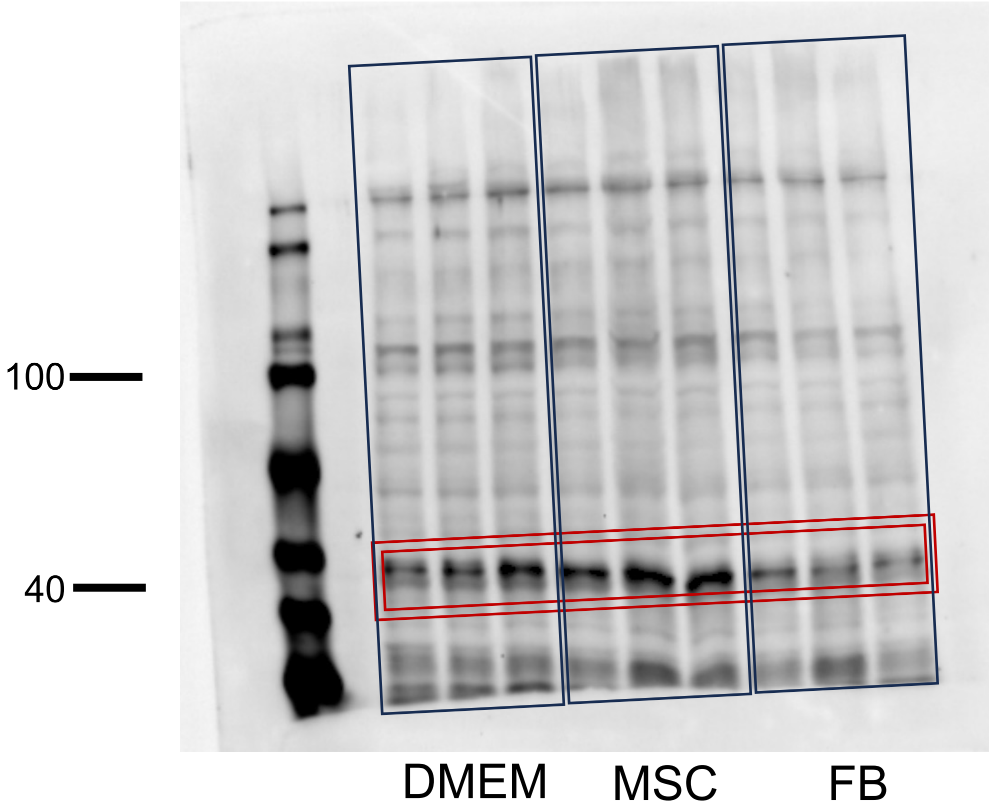

Stain-Free-Image

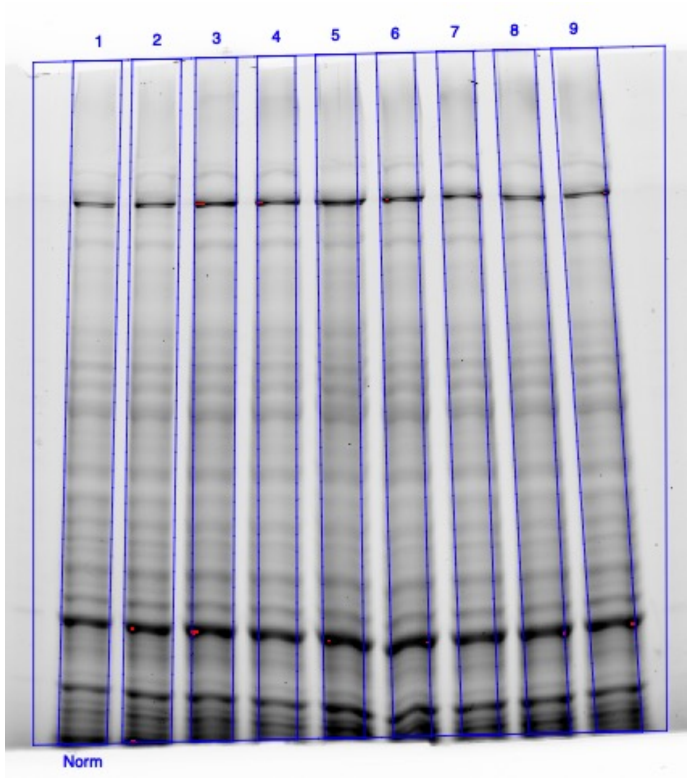

CXCR-4 VMCUB-1

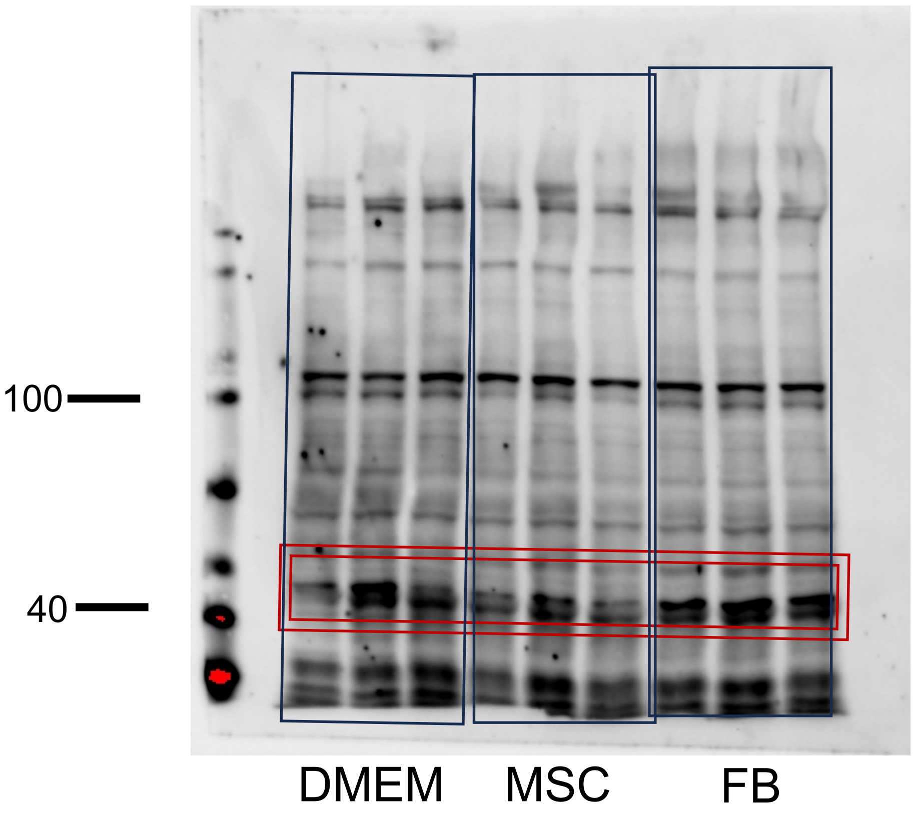

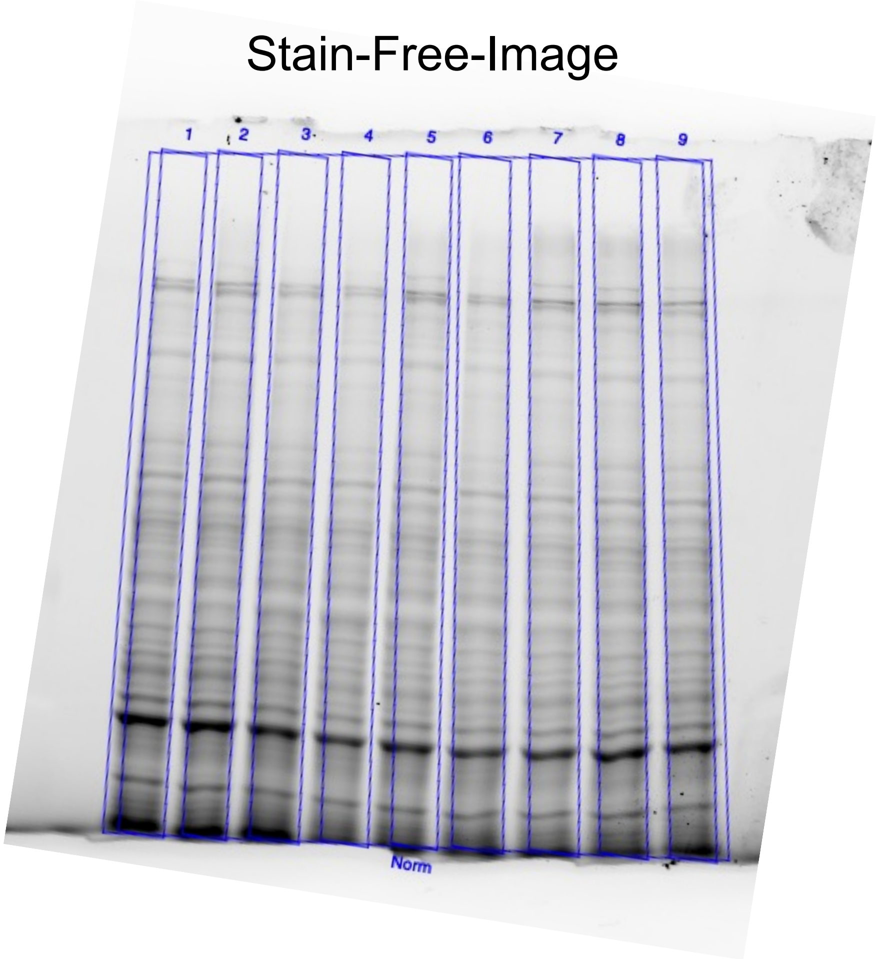

CXCR-4 UMUC-3

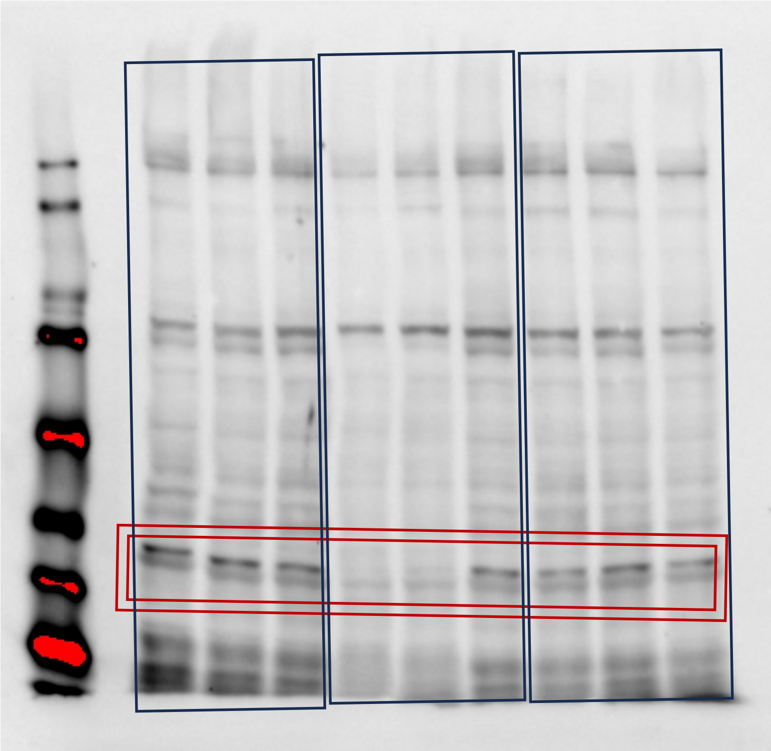

Stain-Free-Image

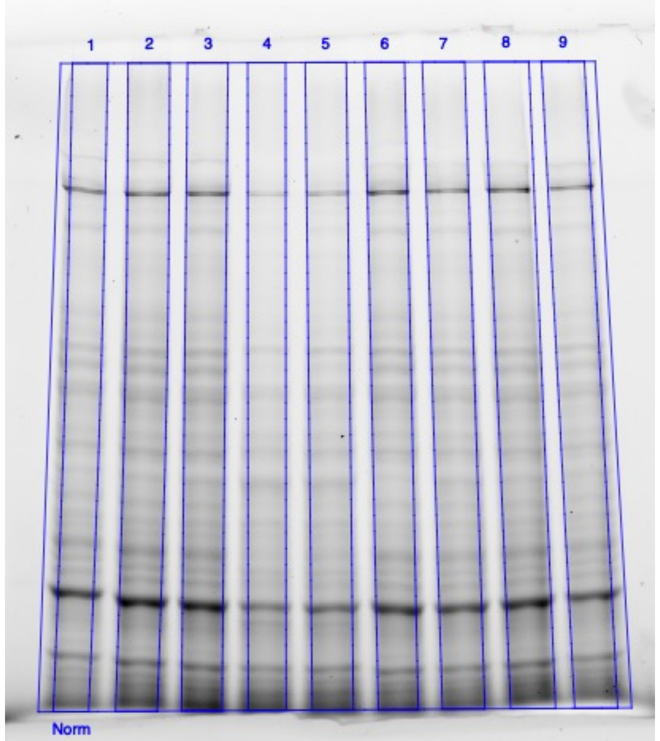

SMAD4 BFTC-905

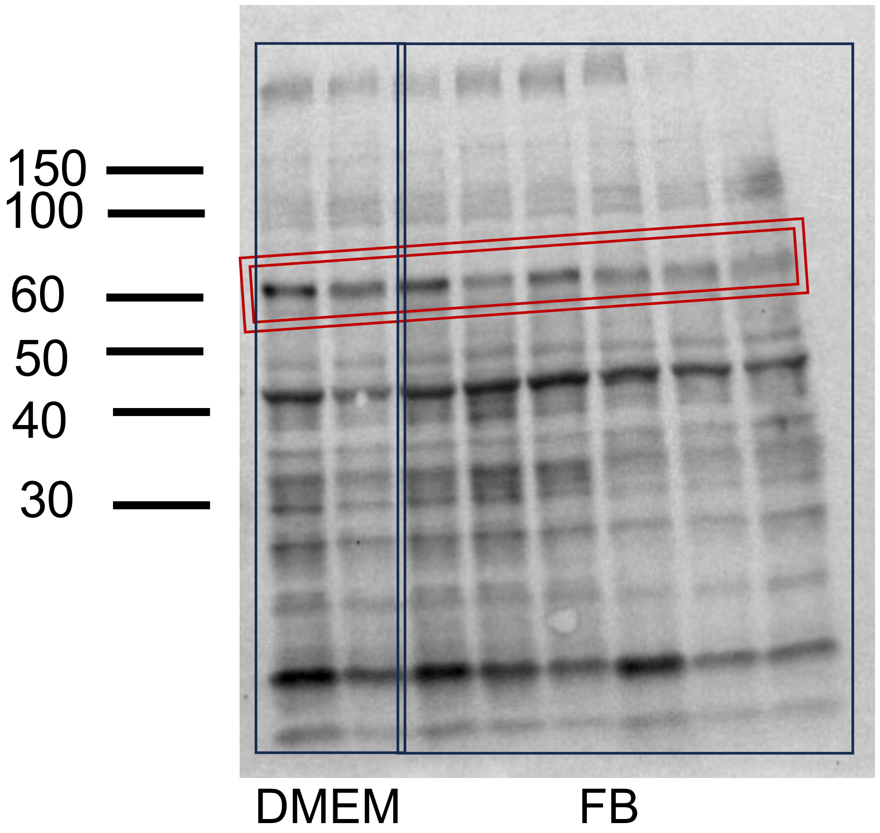

Stain-Free-Image

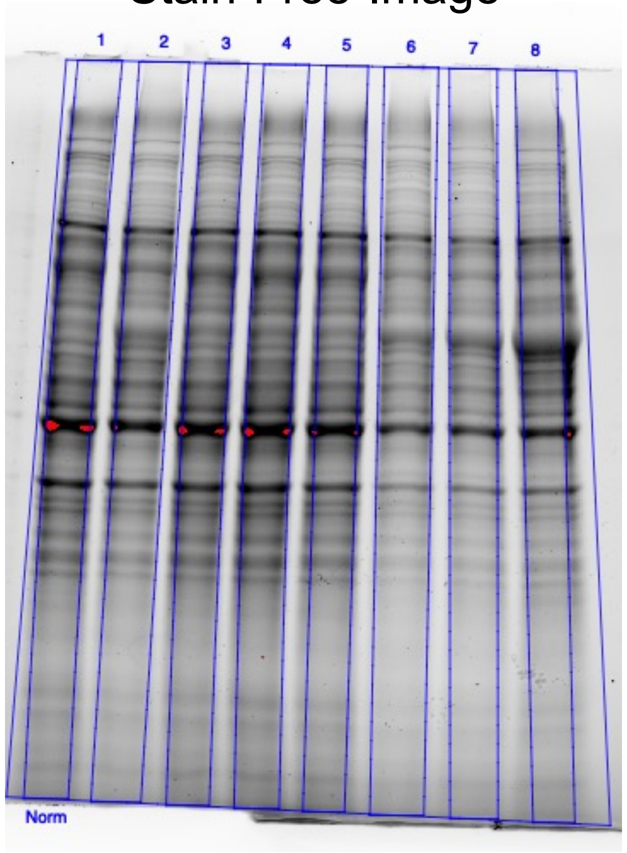

SMAD4 BFTC-905

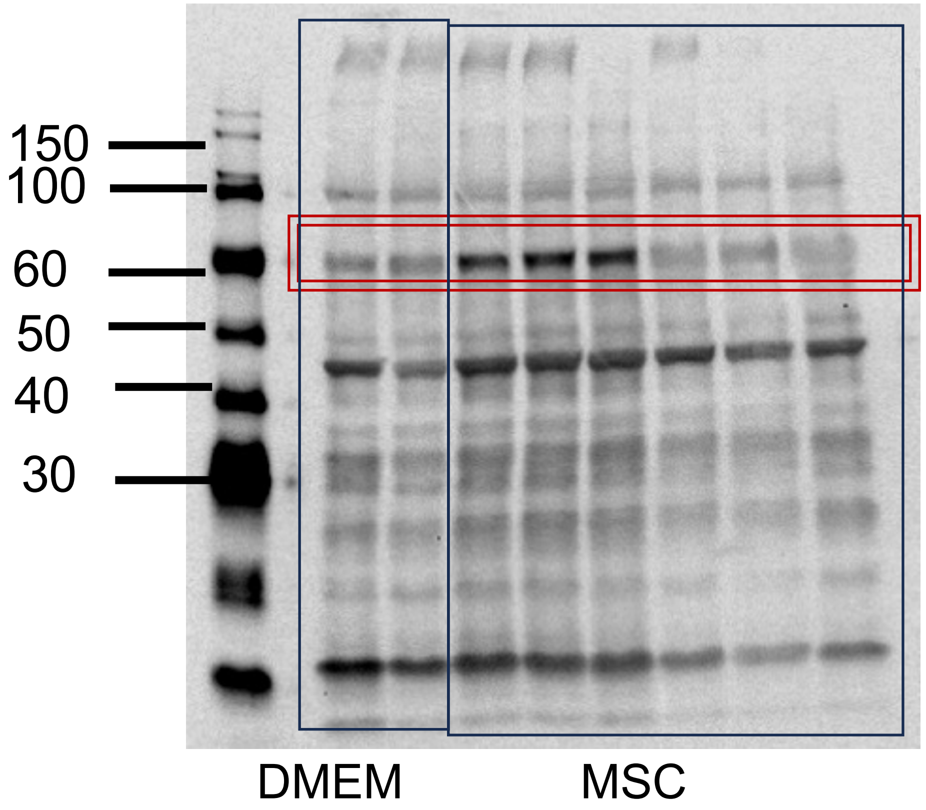

Stain-Free-Image

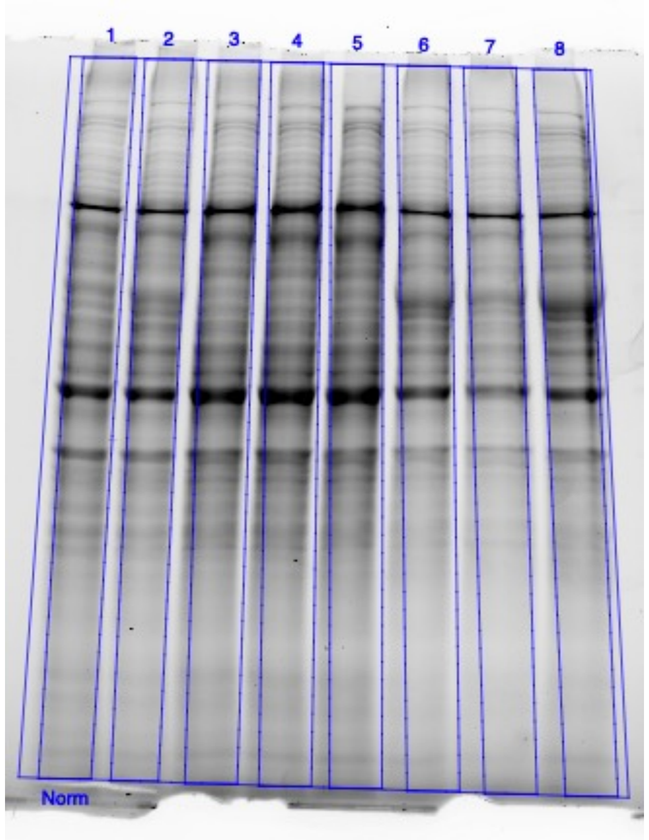

SMAD4 VM-CUB1

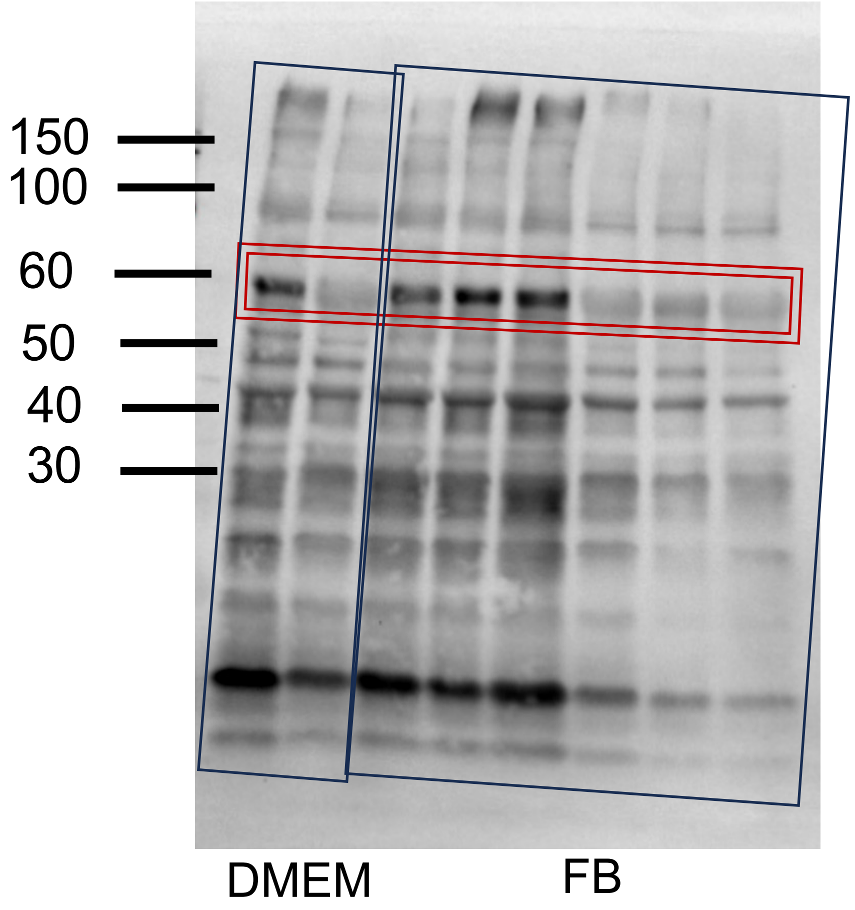

Stain-Free-Image

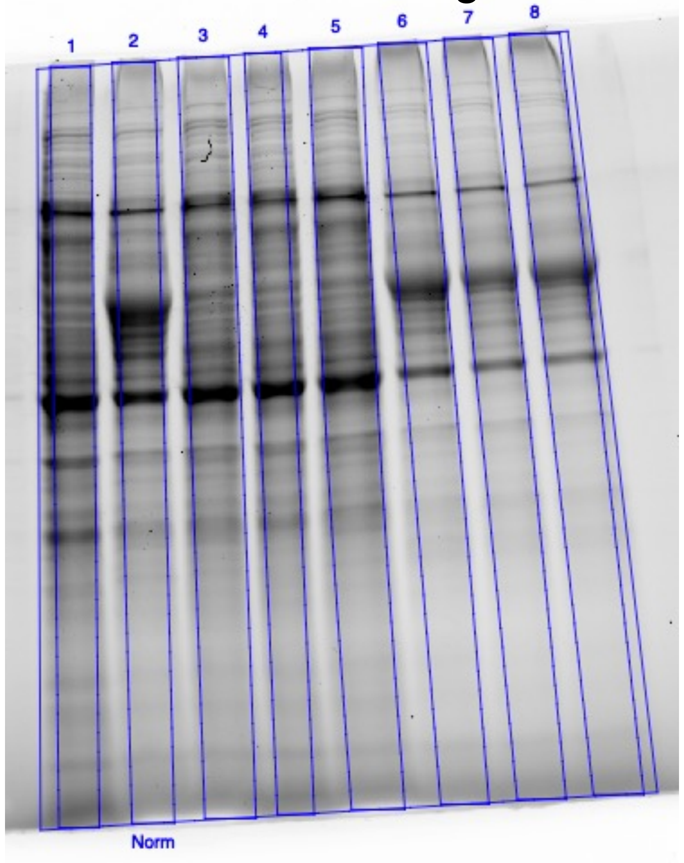

SMAD4 VMCUB-1

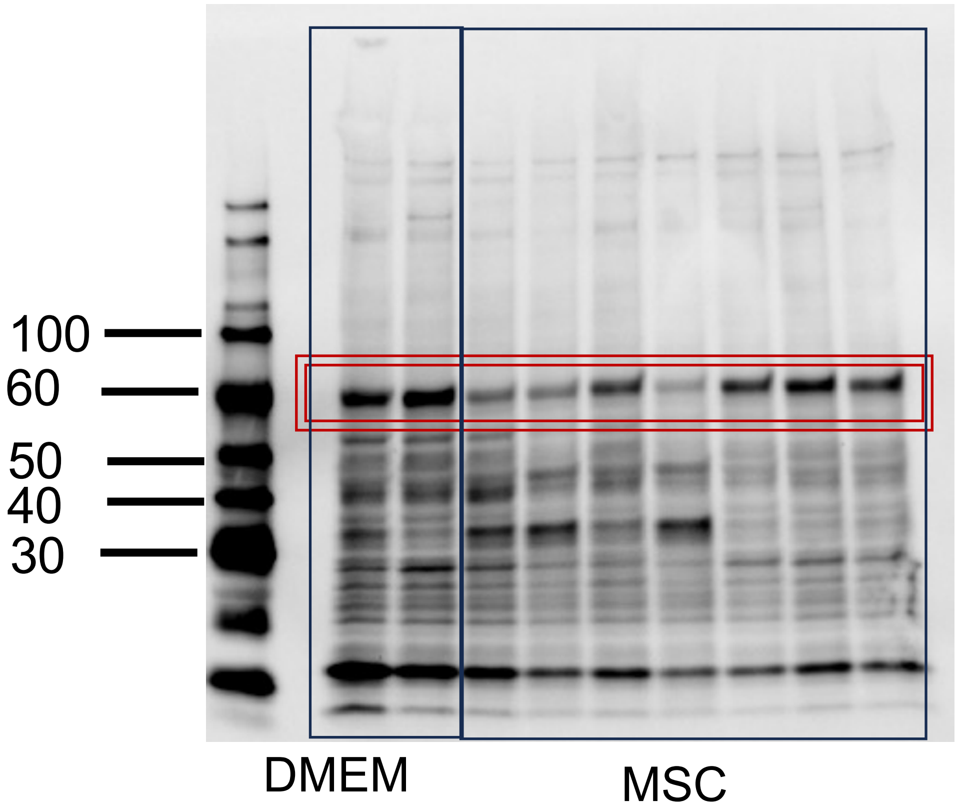

Stain-Free-Image

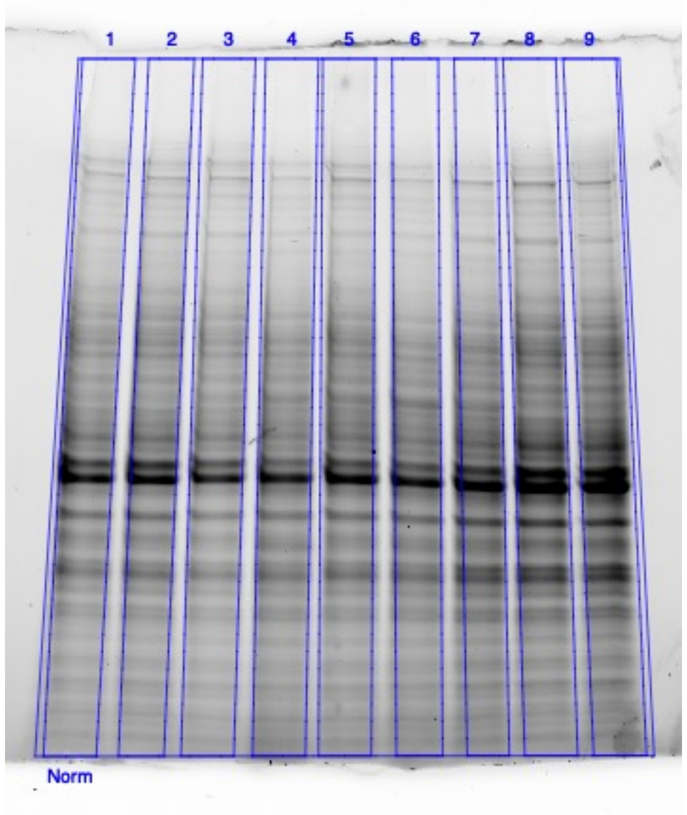

SMAD4 UMUC-3

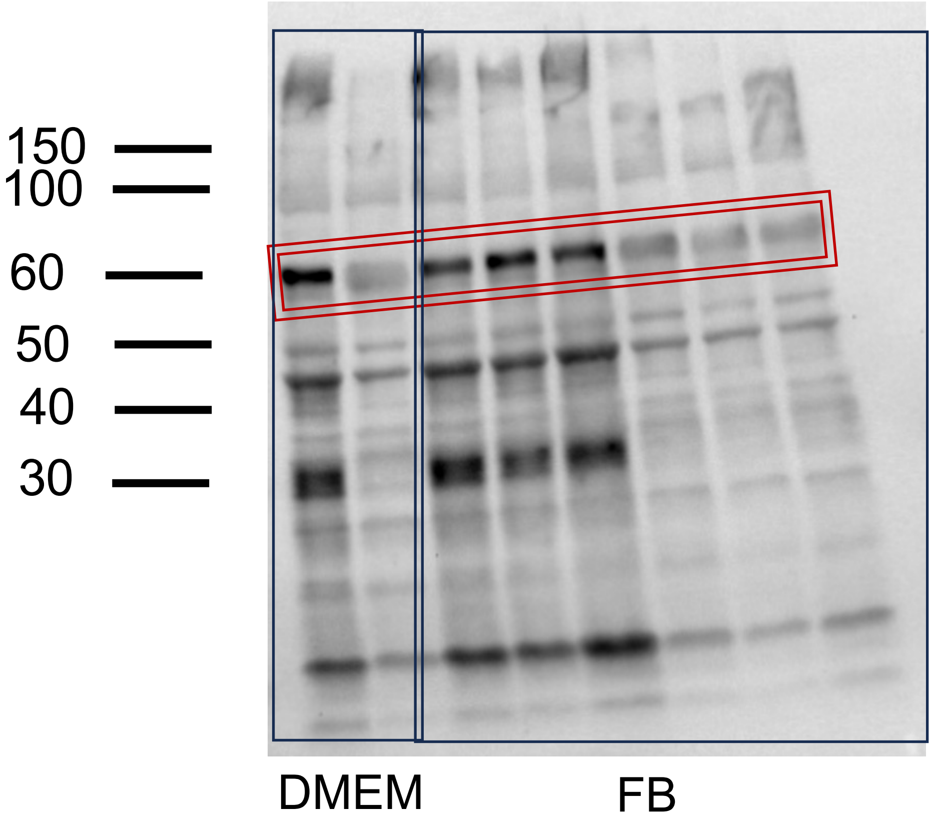

Stain-Free-Image

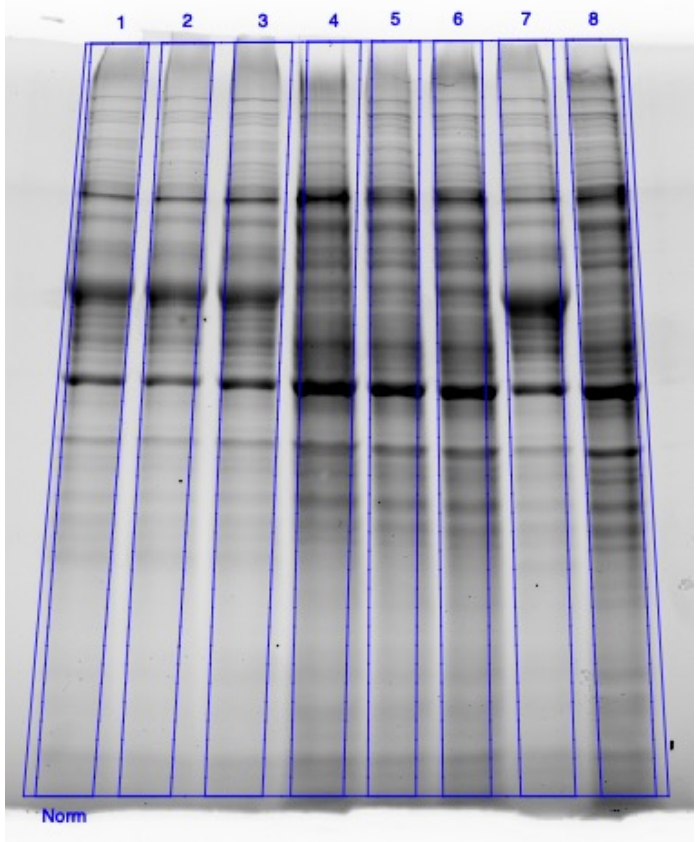

SMAD4 UMUC-3

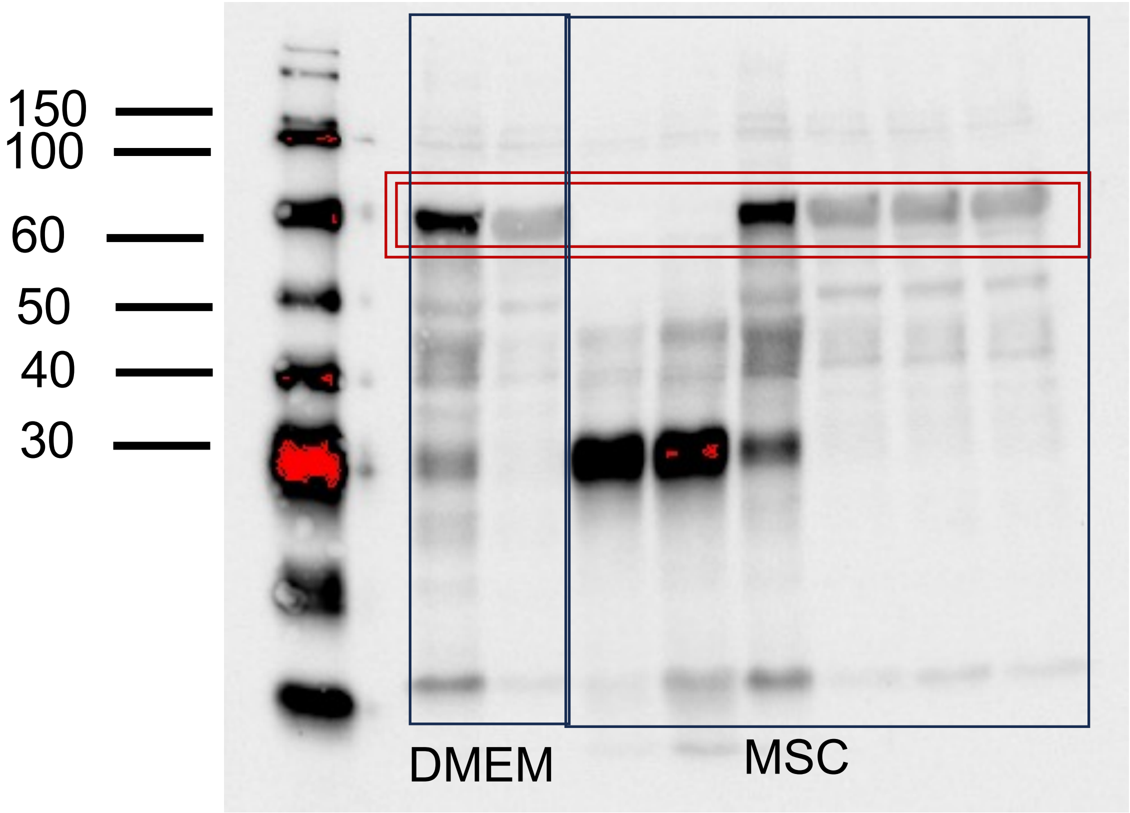

Stain-Free-Image

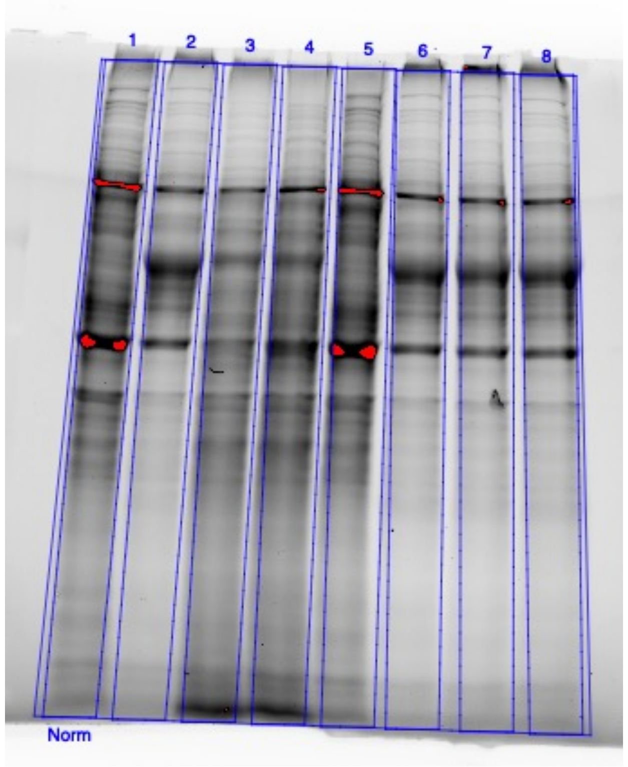

α-SMA BFTC-905

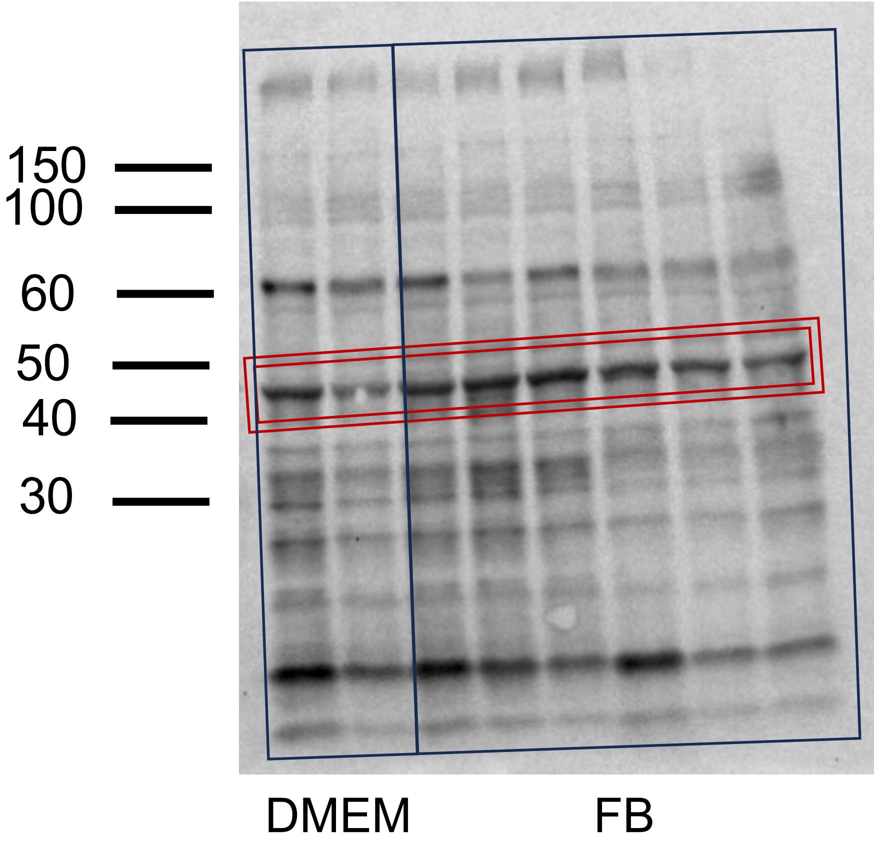

Stain-Free-Image

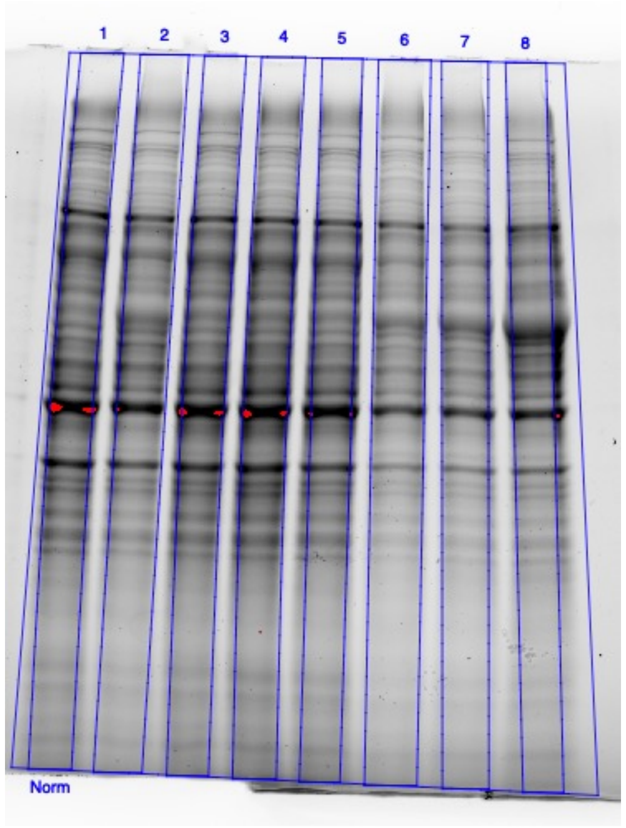

α-SMA BFTC-905

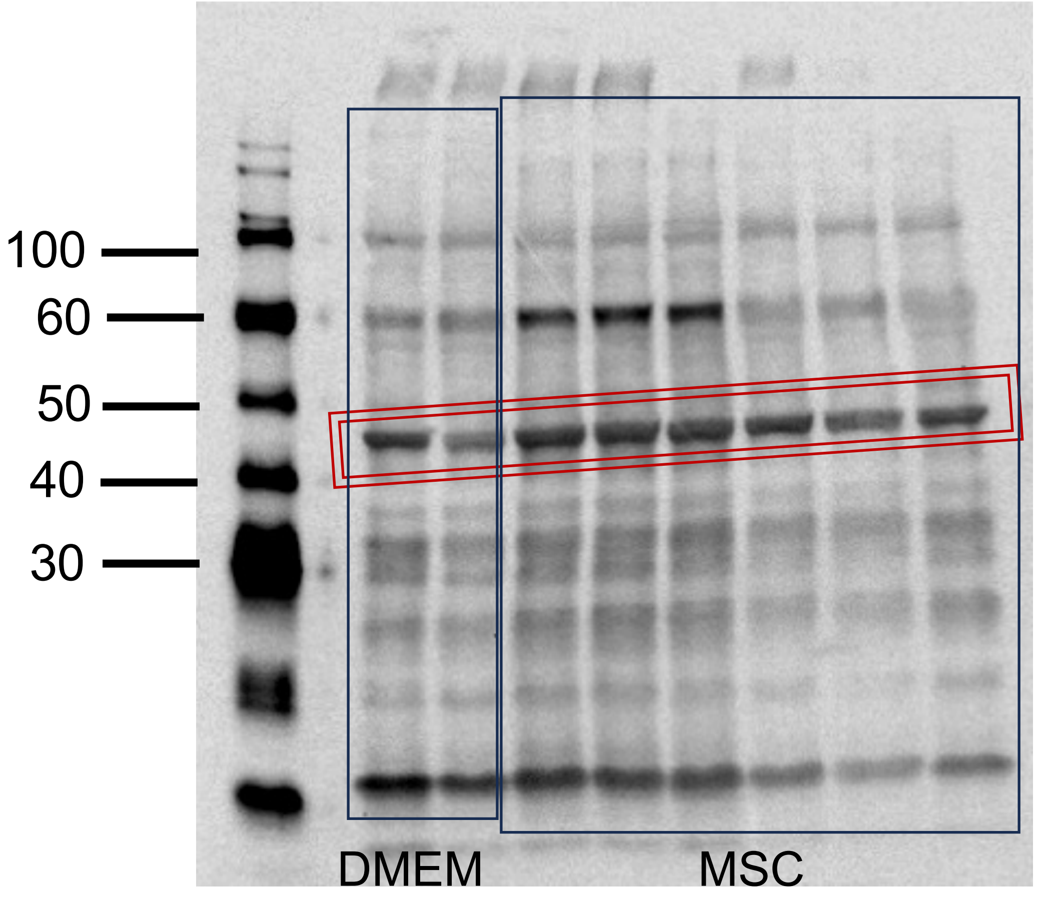

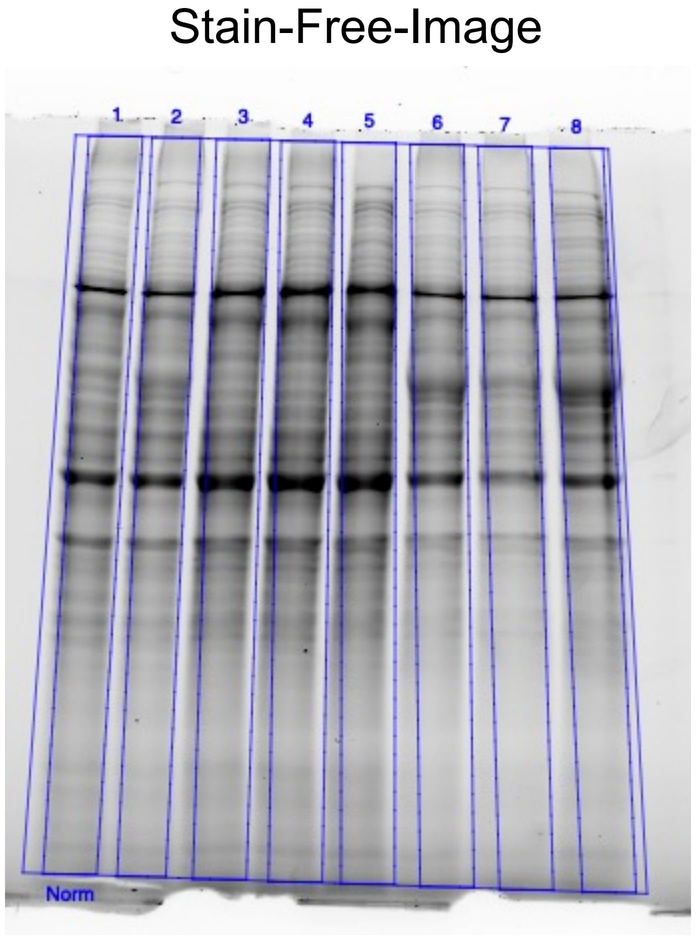

α-SMA VMCUB-1

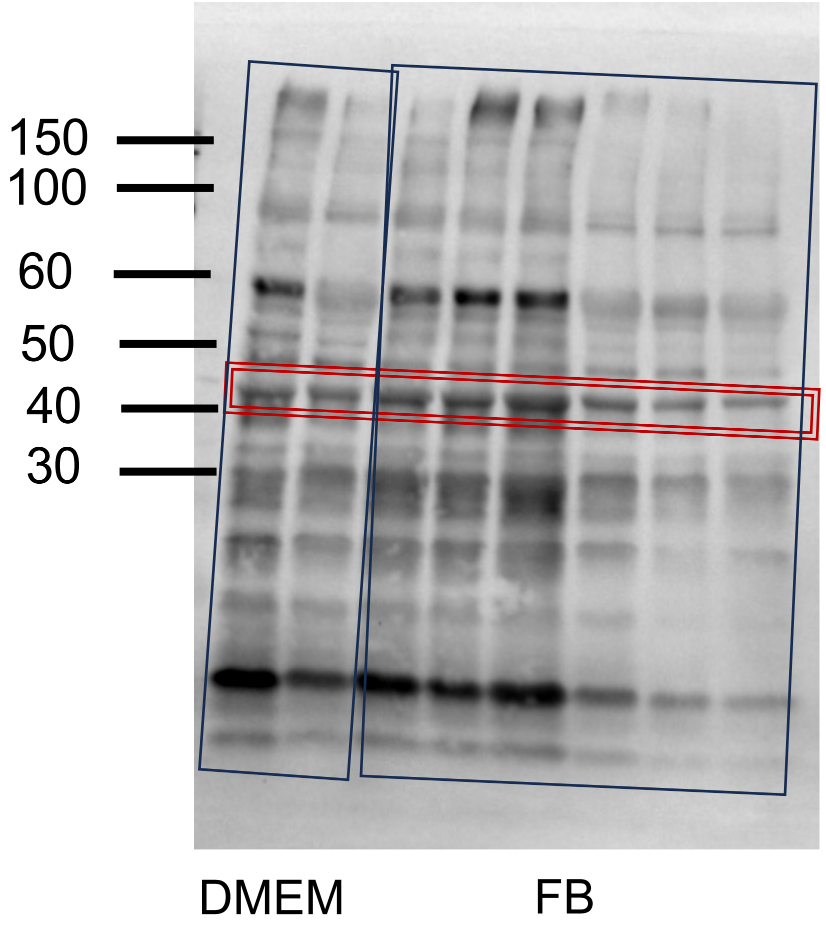

Stain-Free-Image

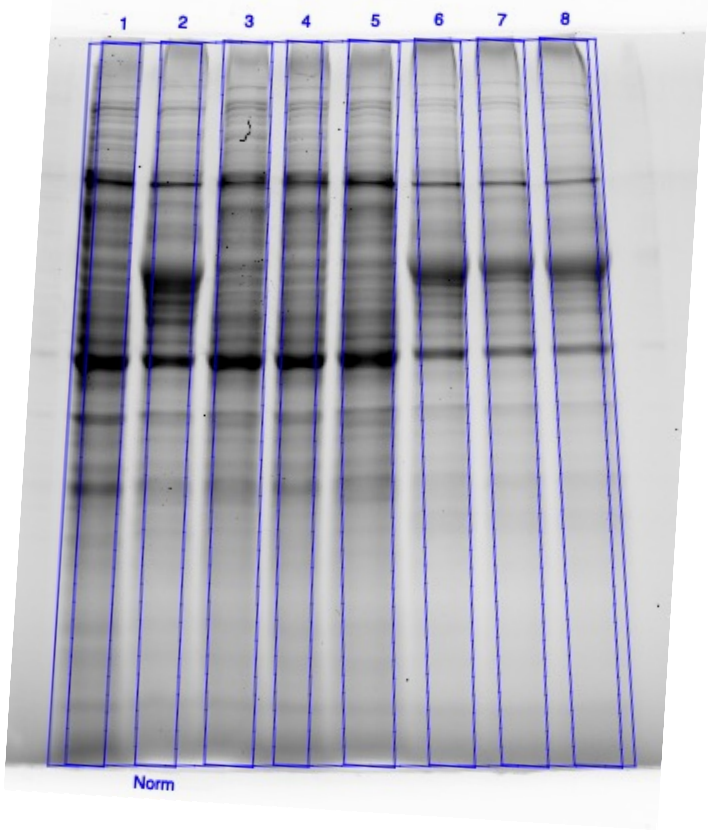

α-SMA VMCUB-1

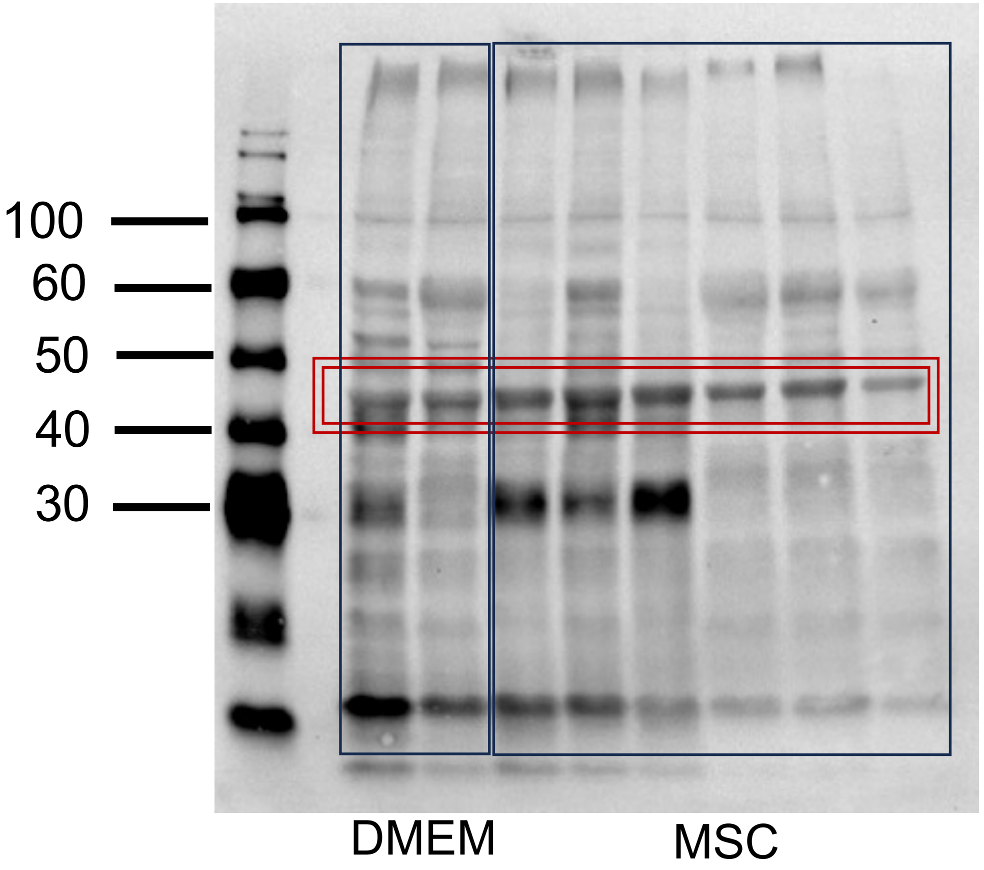

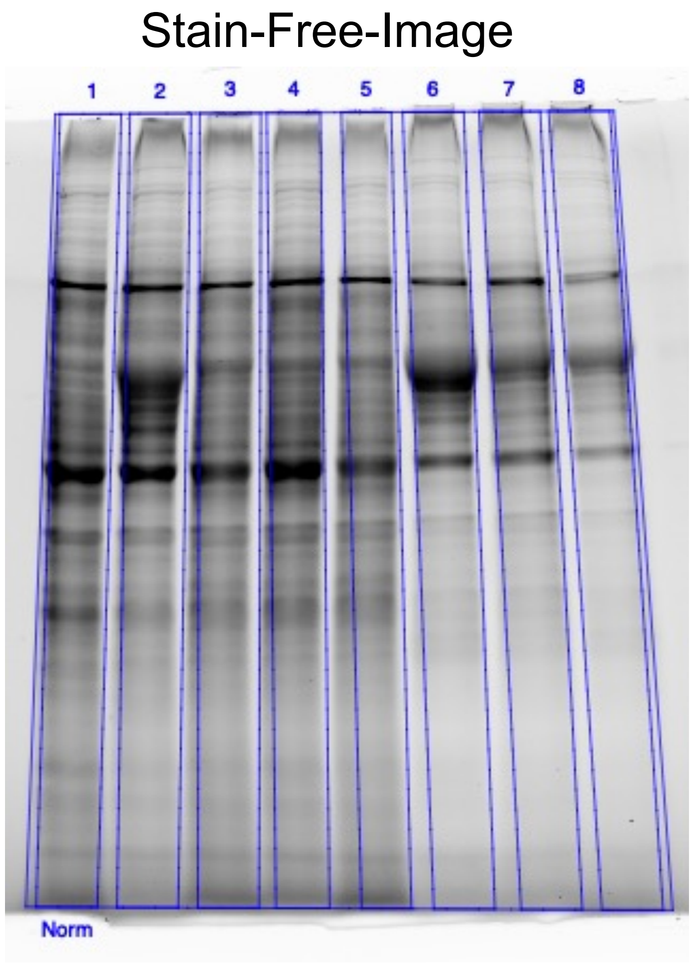

α-SMA UMUC-3

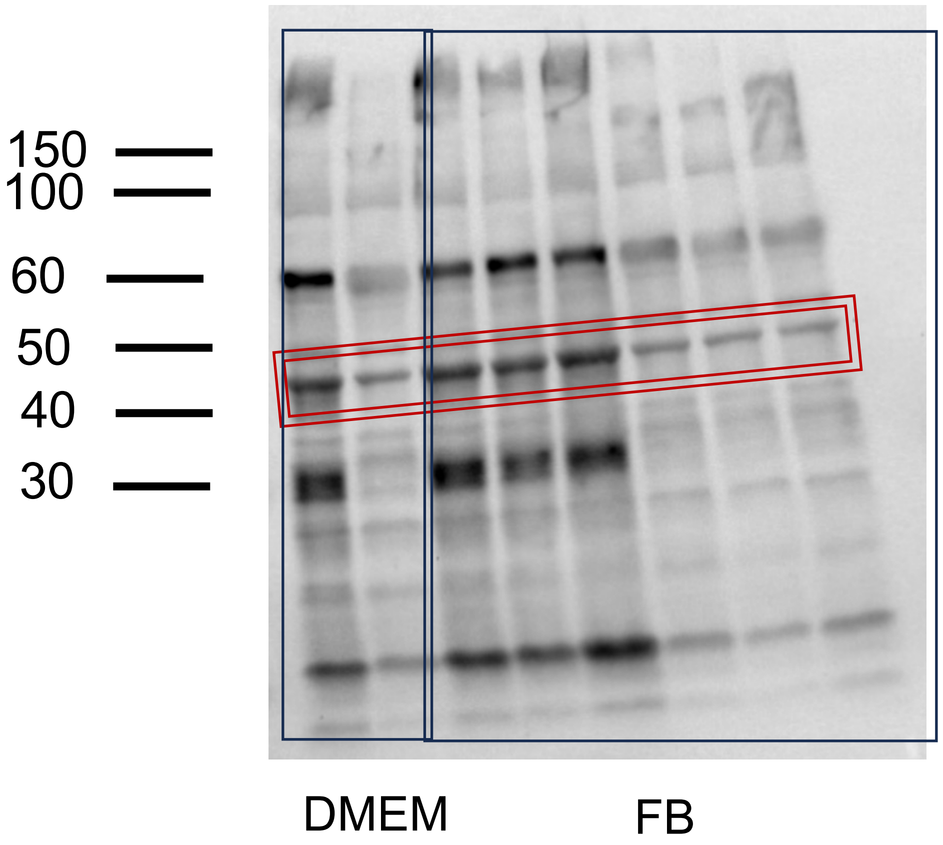

Stain-Free-Image

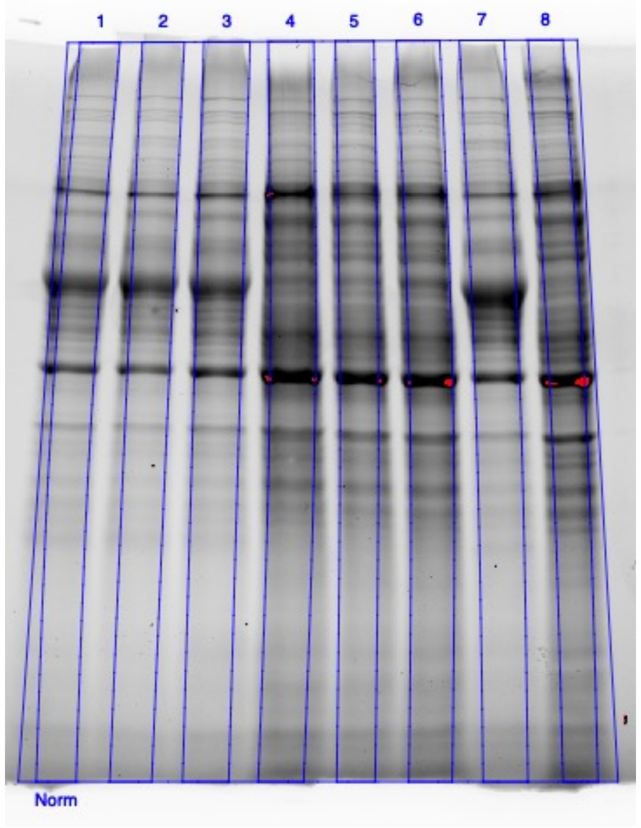

α-SMA UMUC-3

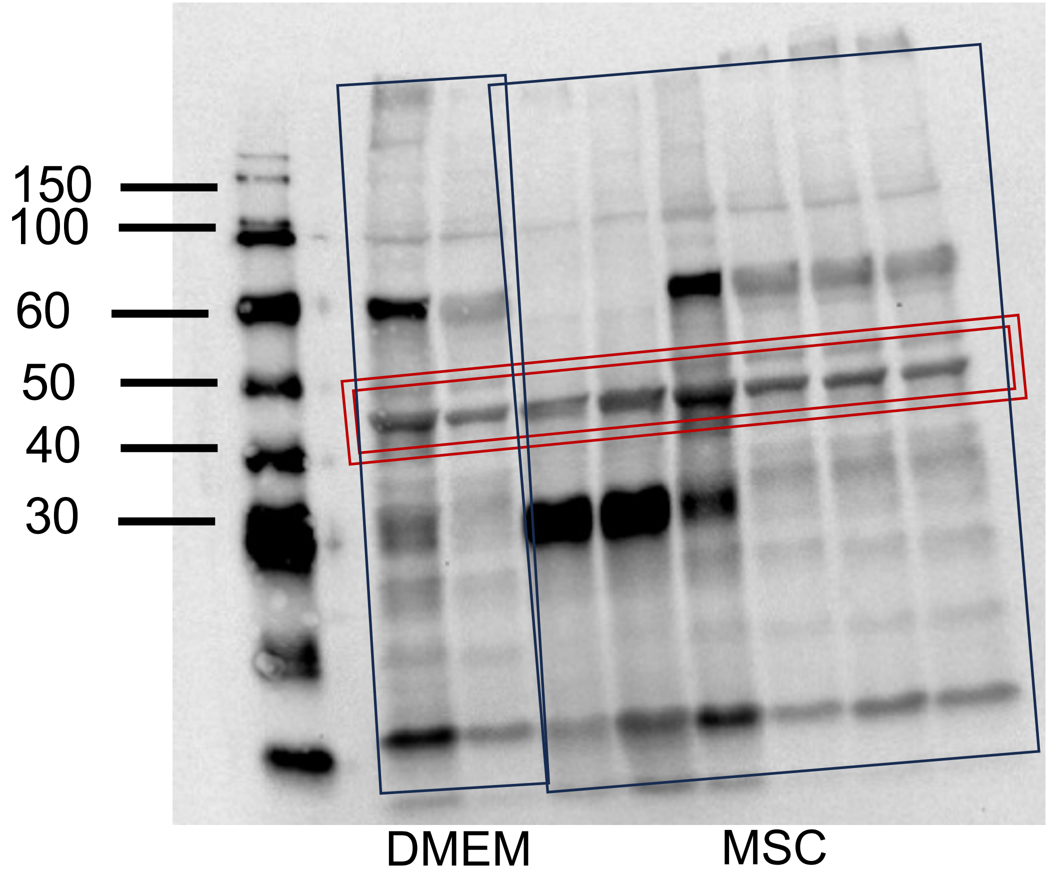

Stain-Free-Image

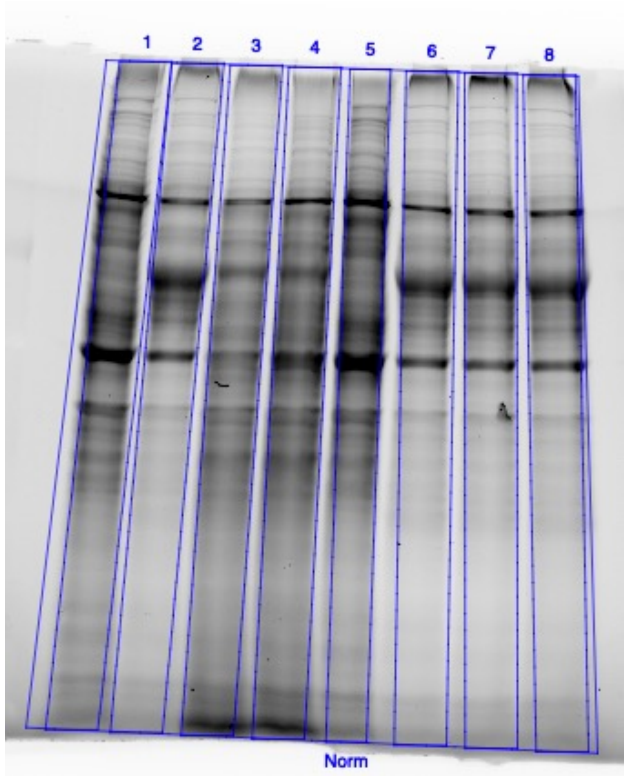

E-cadherin BFTC-905

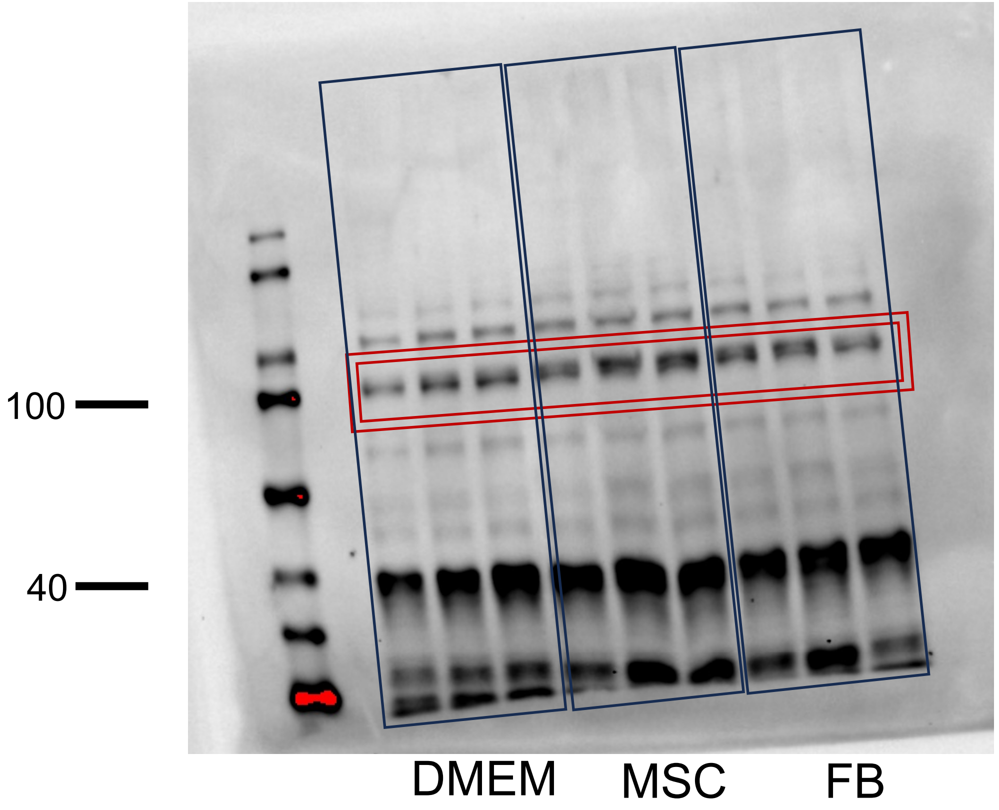

Stainfree-Blot

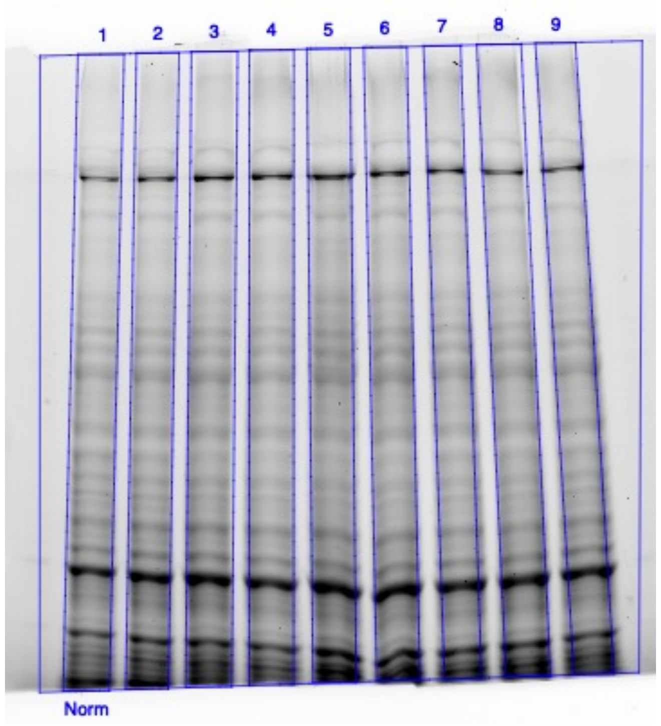

Vimentin BFTC-905

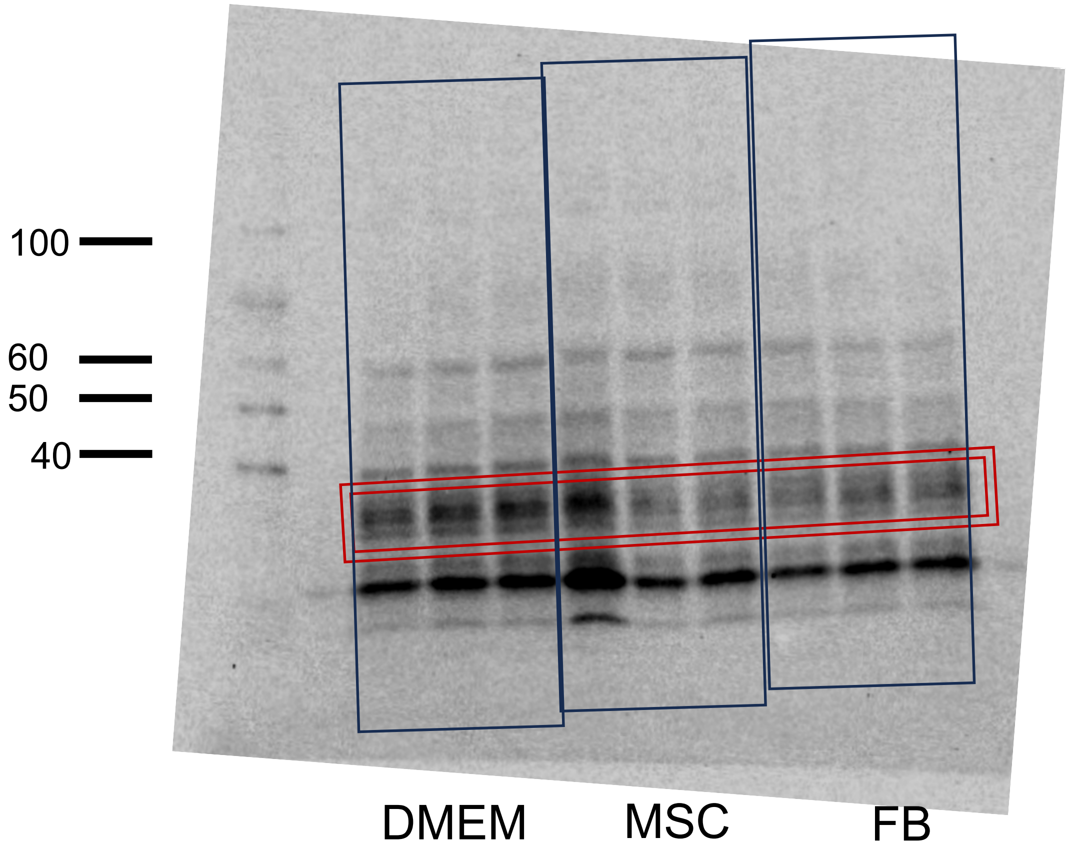

Stainfree-Blot

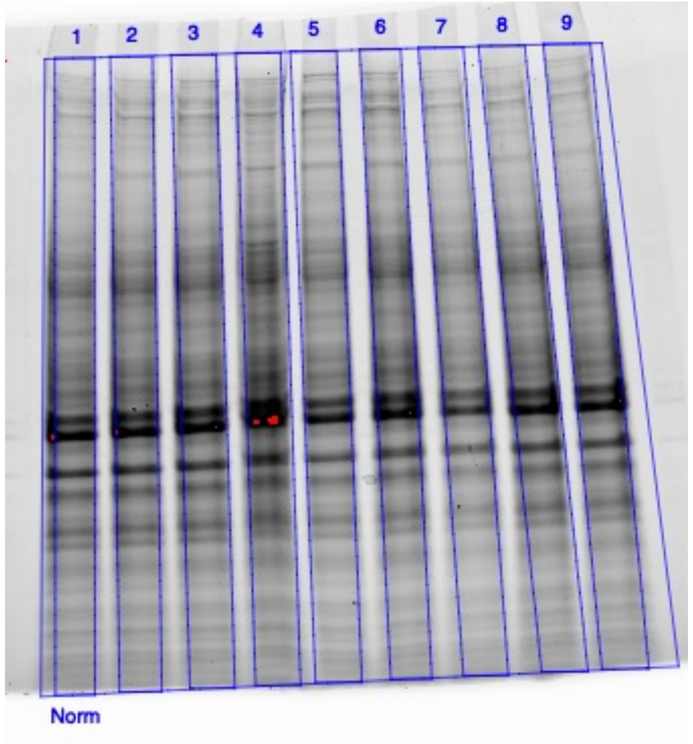

E-cadherin VMCUB-1

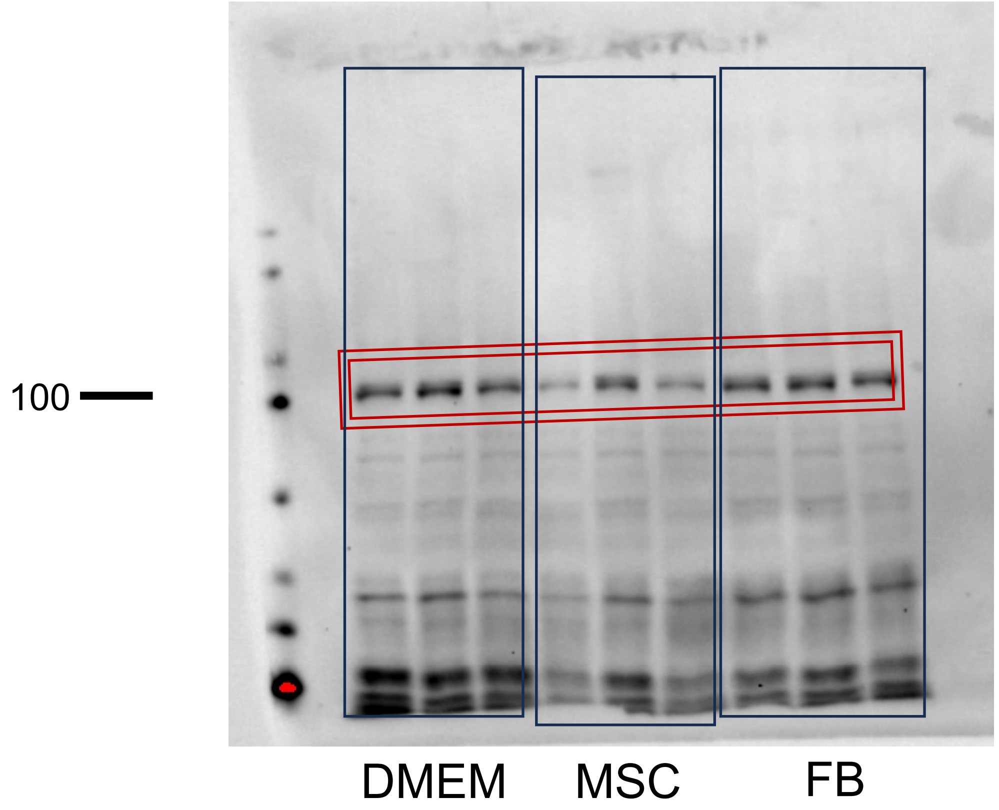

Stainfree-Blot

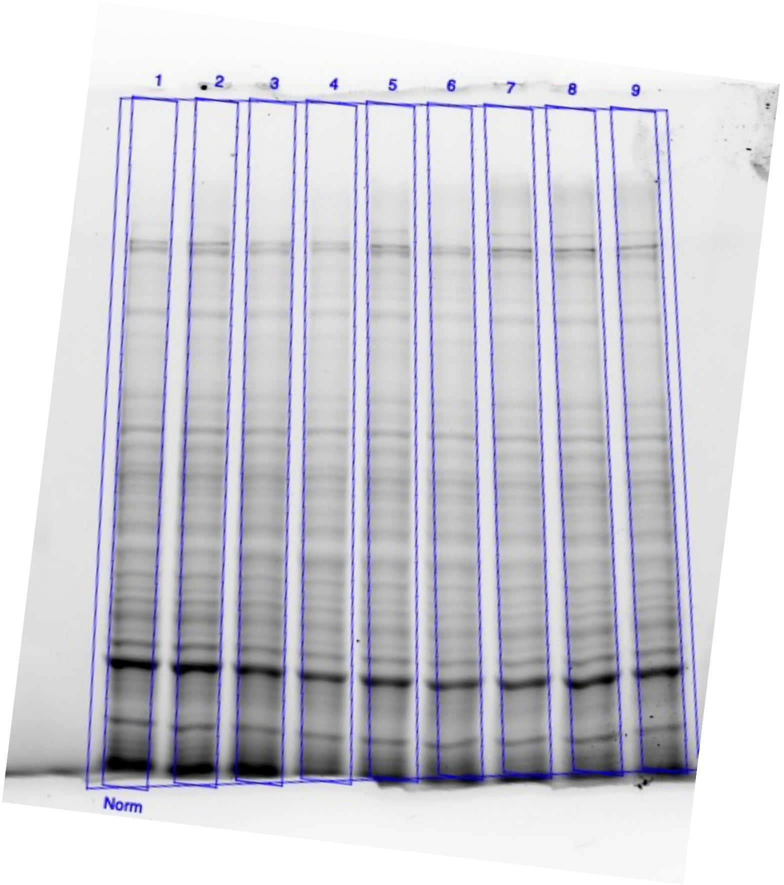

Vimentin VMCUB-1

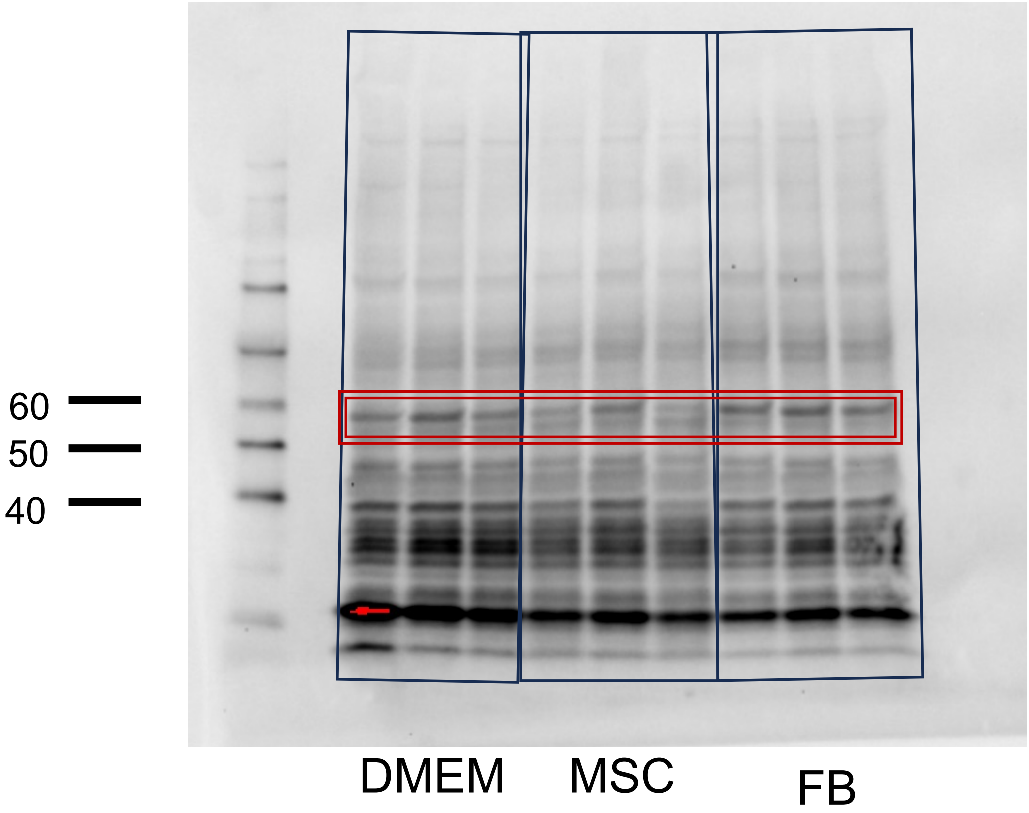

Stainfree-Blot

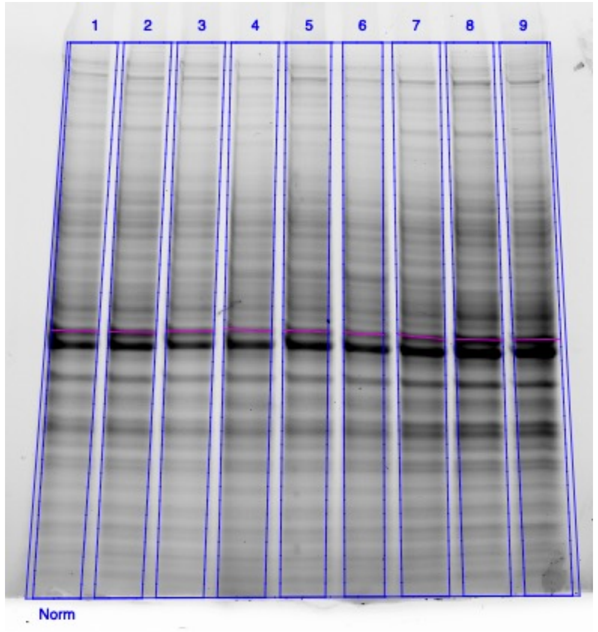

E-cadherin UMUC-3

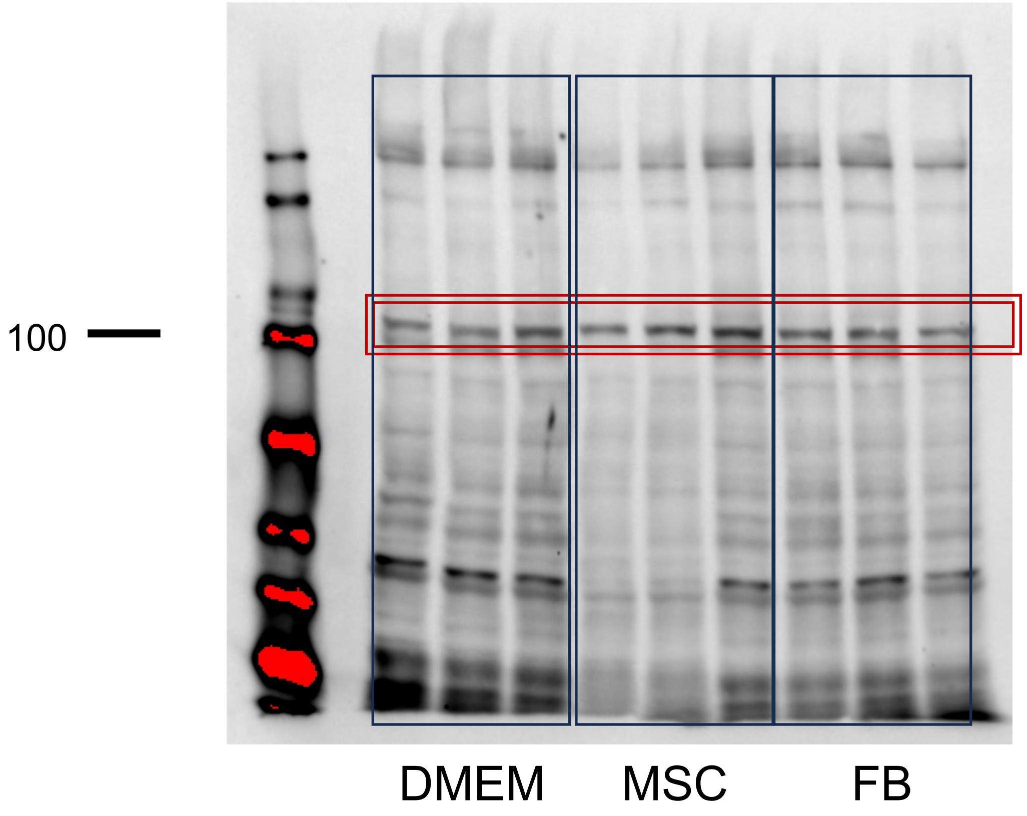

Stainfree-Blot

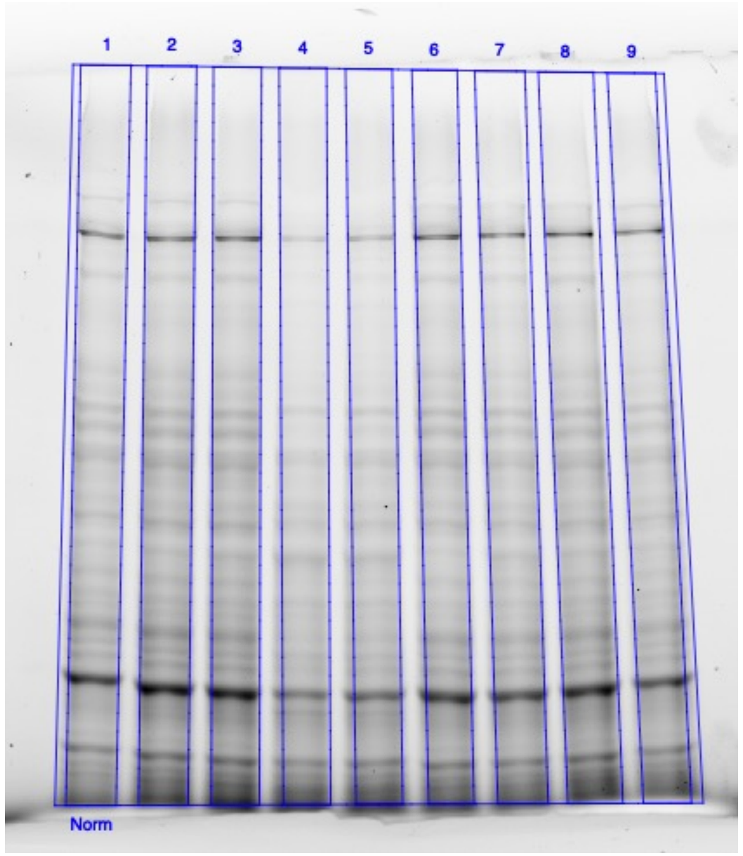

Vimentin UMUC-3

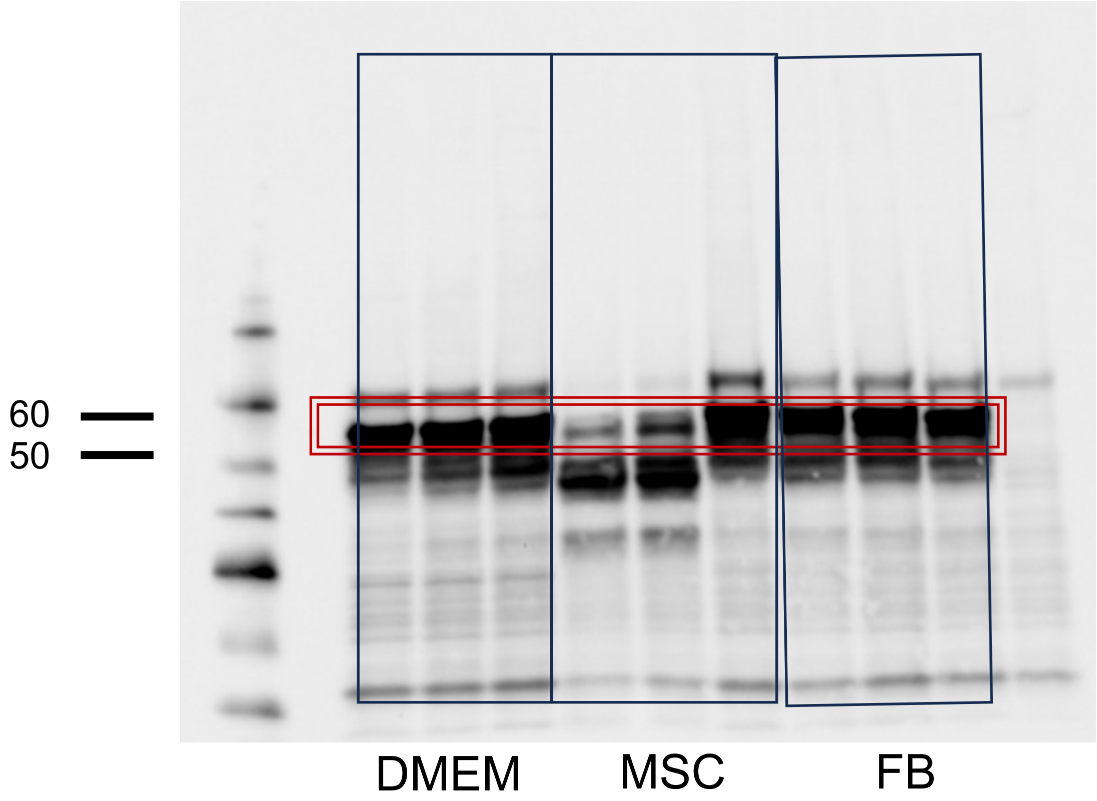

Stainfree-Blot

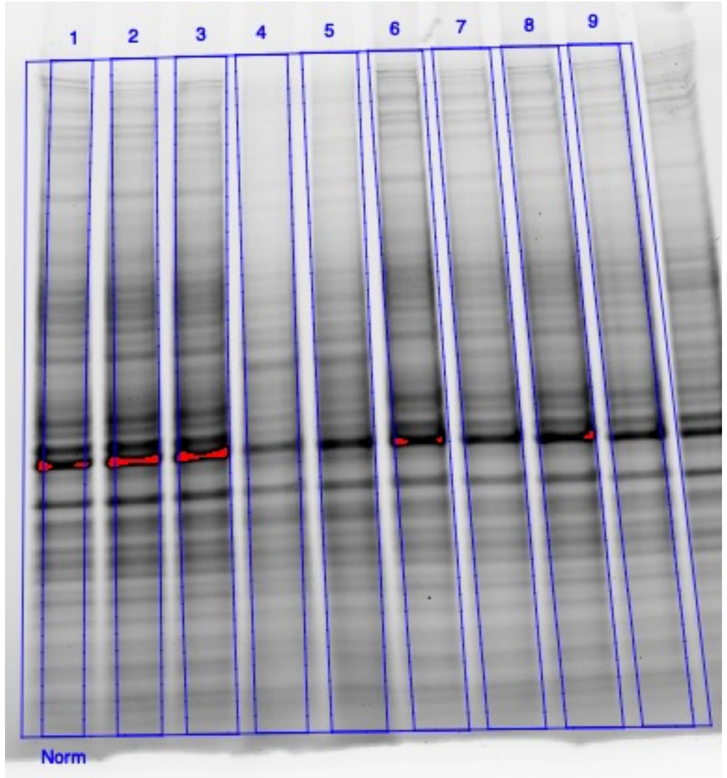

Supplement: Additional File 5 — Lists of common DEGs corresponding with the analyses displayed in . [file DataSheet_1.pdf]
