# Supplementary material for: Tumorigenic effects of human mesenchymal stromal cells and fibroblasts on bladder cancer cells
Source: Front Oncol. 2023 Sep 13;13:1228185. doi: 10.3389/fonc.2023.1228185 (PMC10534007; doi:10.3389/fonc.2023.1228185)
Supplement: Additional File 6 — qRT-PCR analysis of anti-apoptotic regulators. Gene expression changes in UCC were measured by quantitative RT-PCR. Tata-box binding protein (TBP) was used as a reference gene. Gene expression changes in cells treated with indicated CM were normalized to the respective DMEM controls. [file DataSheet_2.pdf]

Proteins secreted in CM	Function	Effect on (bladder) cancer
**Thrombospondin-1**	• activates TGF-β and mediates cell-to-cell and cell-to-matrix interactions<br>• can bind to fibrinogen, fibronectin, laminin, type V collagen and integrins α-V/β-1<br><br>HGNC:HGNC:11785	• display both oncogenic and tumor-suppressive qualities<br>• involved in cell adhesion and spreading, an important feature of wound healing as well as cancer cell migration<br>• previously known for the activation of TGF-β to promote tumor growth and inflammation [44]
**Serpin E1/PAI**	• TGF-β responsive element<br>• principal inhibitor of tissue plasminogen activator (tPA) and urokinase (uPA)<br>• impact on both proteolytic activity and cell migration during angiogenesis<br><br>HGNC:HGNC:8583	• controversial<br>• PAI-1 has a strong role in cancer angiogenesis<br>• high levels in patients with breast and BC correlates with poor survival [47]
**IL-6**	• inflammation and maturation of B cells<br>• pyrogen<br><br>HGNC:HGNC:6018	• Il-6 induced EMT in BC [58]<br>• IL-6 support CD44 expression in BC [59]
**CXCL8/IL-8**	• major mediator of inflammatory response<br>• chemotactic factor<br><br>HGNC:HGNC:6025	• IL-8 increased invasiveness, metastasis and angiogenesis in UCC [60]
**CCL2/MCP-1**	• involved in immunoregulatory and inflammatory processes<br>• chemotactic activity<br><br>HGNC:HGNC:10618	• suppressed CCL2 inhibits proliferation and invasion of BC cells [61]
**Progranulin**	• regulate cell growth, normal development, wound healing<br><br>HGNC:HGNC:4601	• Progranulin modulates BC cell motility and invasion [62]<br>• suppression of progranulin sensitizes BC cells to cisplatin [63]
**Osteopontin**	• this protein is also a cytokine that upregulates expression	• Osteopontin accelerates metastasis in BC [64]

	of Interferon (IFN)-γ and IL-12<br><br>HGNC:HGNC:11255	
**SPARC/Osteonectin**	• required for the collagen in bone to become calcified but is also involved in extracellular matrix synthesis<br><br>HGNC:HGNC:11219	• inhibits invasion and metastasis [65, 66]
**Dkk-1**	• inhibits WNT-signaling, important in adults bone formation<br><br>HGNC:HGNC:2891	• elevated in serum of BC patients, correlated with metastasis [67]<br>• in several malignancies Dkk-1 promotes tumor progression through Wnt-dependent and independent pathway [68]
**MMP-2**	• breakdown of extracellular matrix, type IV collagenase<br><br>HGNC:HGNC:7166	• tumor invasion of bladder cells can be driven by MMP-2 [69]
**MMP-3**	• breakdown of extracellular matrix<br>• degrades fibronectin, laminin, collagens III, IV, IX, and X, and cartilage proteoglycans<br><br>HGNC:HGNC:7173	• elevated serum levels in BC patients are associated with recurrence [70]
**Axl**	• involved in several cellular functions including growth, migration, aggregation and anti-inflammation in multiple cell types<br><br>HGNC:HGNC:905	• Axl depletion has a minor effect on BC cell viability [74]
**Decorin**	• inhibitor of TGF-β<br>• effect on autophagy and inflammation<br>• inhibitory effect on angiogenesis and tumorigenesis<br>• antagonizes IGF-receptor function<br>• a role in collagen fibril assembly<br><br>HGNC:HGNC:2705	• Decorin induces cell cycle arrest and apoptosis have antimetastatic and antiangiogenic properties in TME [75]<br>• inhibits proliferation in BC cells [76]

**Lumican**	• Lumican may regulate collagen fibril organization<br><br>HGNC:HGNC:6724	• Lumican inhibits migration and invasion of BC [77]<br>• seems to be tumor specific [78]
**Tenascin C**	• extracellular matrix protein<br><br>HGNC:HGNC:5318	• increased stromal expression associated with worse overall survival; tumor cell expression associated with improved overall survival (Brunner et al. 2004)

Table 1 Cytokines and proteins contained in CM
